# Supplementary figures and images for: Evaluation of protein kinase D auto-phosphorylation as biomarker for NLRP3 inflammasome activation
Source: PLoS One. 2021 Nov 12;16(11):e0248668. doi: 10.1371/journal.pone.0248668 (PMC8589197; doi:10.1371/journal.pone.0248668)

## Slide 1
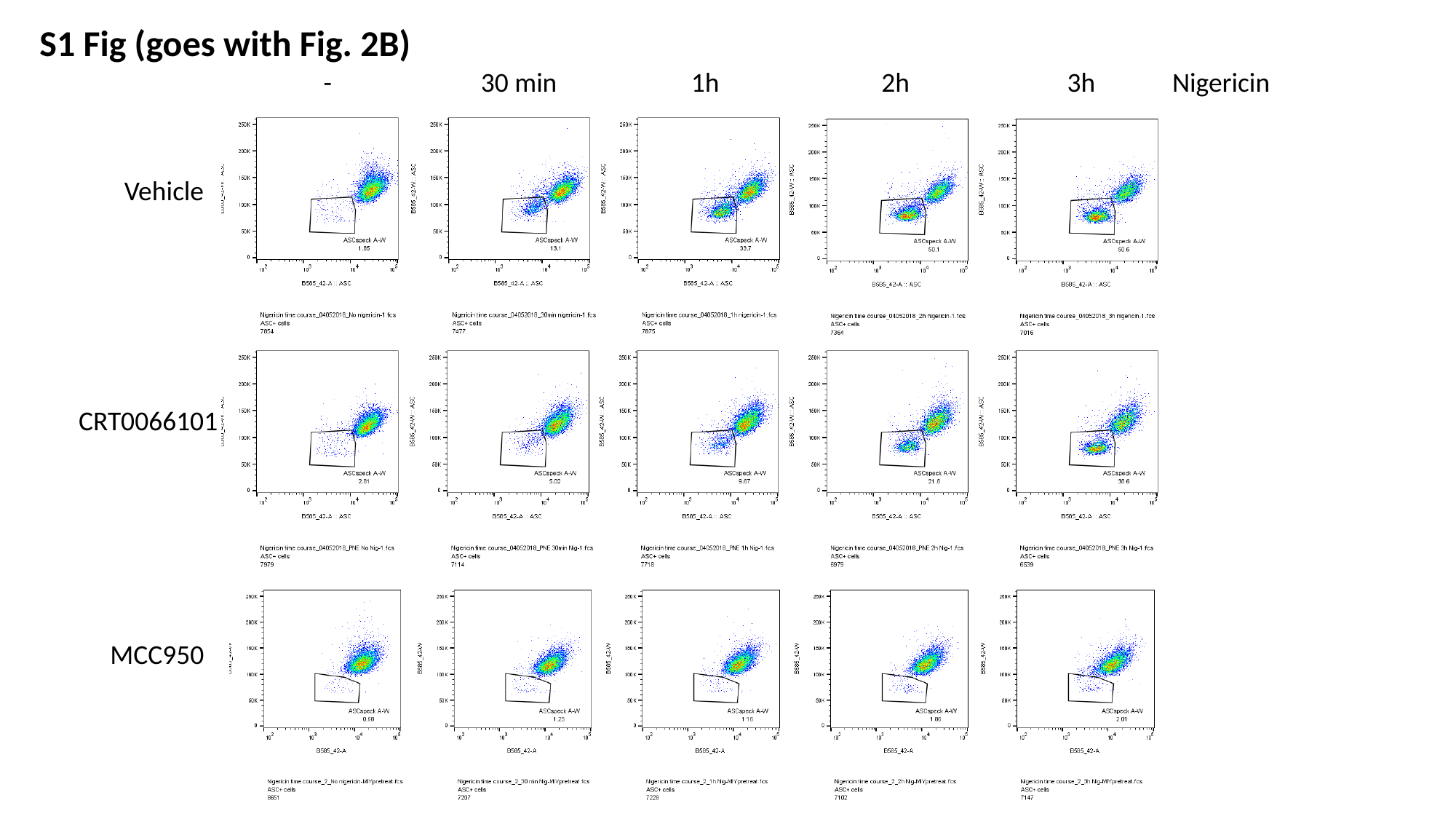

S1 Fig (goes with Fig. 2B)
-
30 min
1h
2h
3h
Nigericin
Vehicle
CRT0066101
MCC950

Supplement: S1 Fig — FACS analysis of ASC speck formation in THP-1 cells treated with 15 uM nigericin for up to 3h (in the presence of 20 μM CGP084892), ± CRT0066101 (10 μM), MCC950 (1 μM), or vehicle control. (PPTX) [file pone.0248668.s001.pptx]
