# Supplementary material for: Evaluation of protein kinase D auto-phosphorylation as biomarker for NLRP3 inflammasome activation
Source: PLoS One. 2021 Nov 12;16(11):e0248668. doi: 10.1371/journal.pone.0248668 (PMC8589197; doi:10.1371/journal.pone.0248668)
Supplement: S1 Raw images — (PPTX) [file pone.0248668.s002.pptx]

## Slide 1
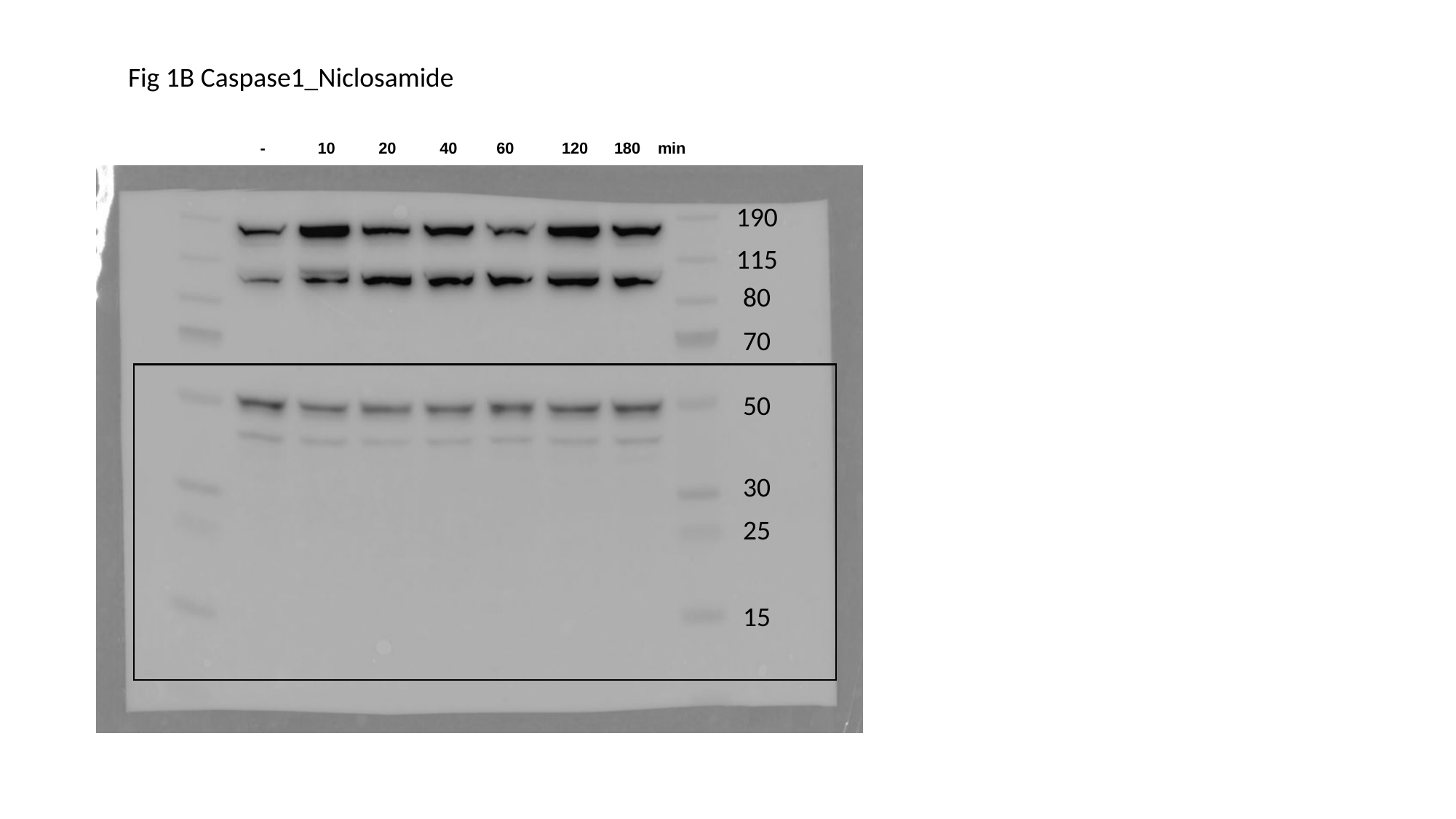

Fig 1B Caspase1_Niclosamide
- 10 20 40 60 120 180 min
190
115
80
70
50
30
25
15

## Slide 2
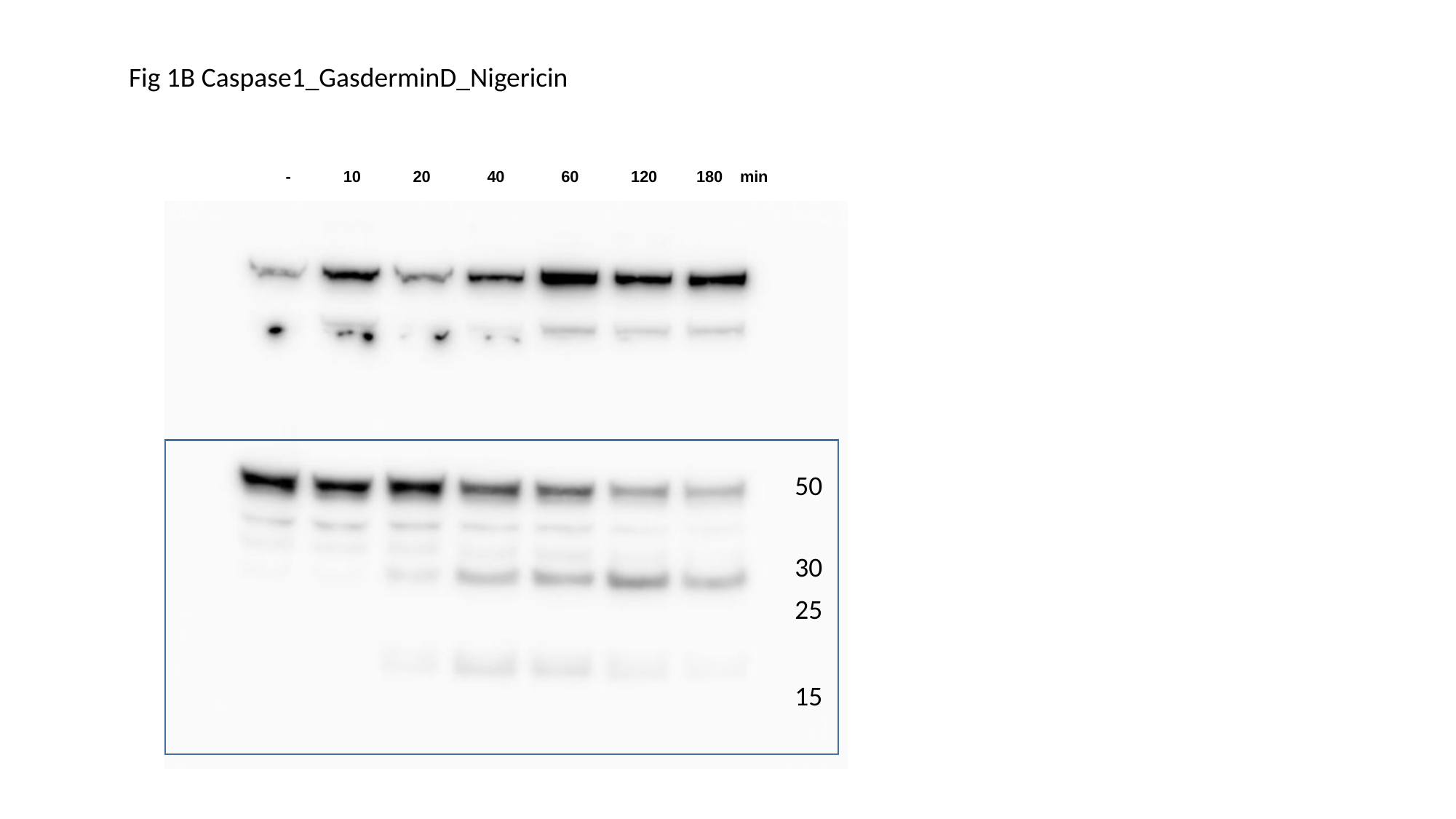

Fig 1B Caspase1_GasderminD_Nigericin
- 10 20 40 60 120 180 min
50
30
25
15

## Slide 3
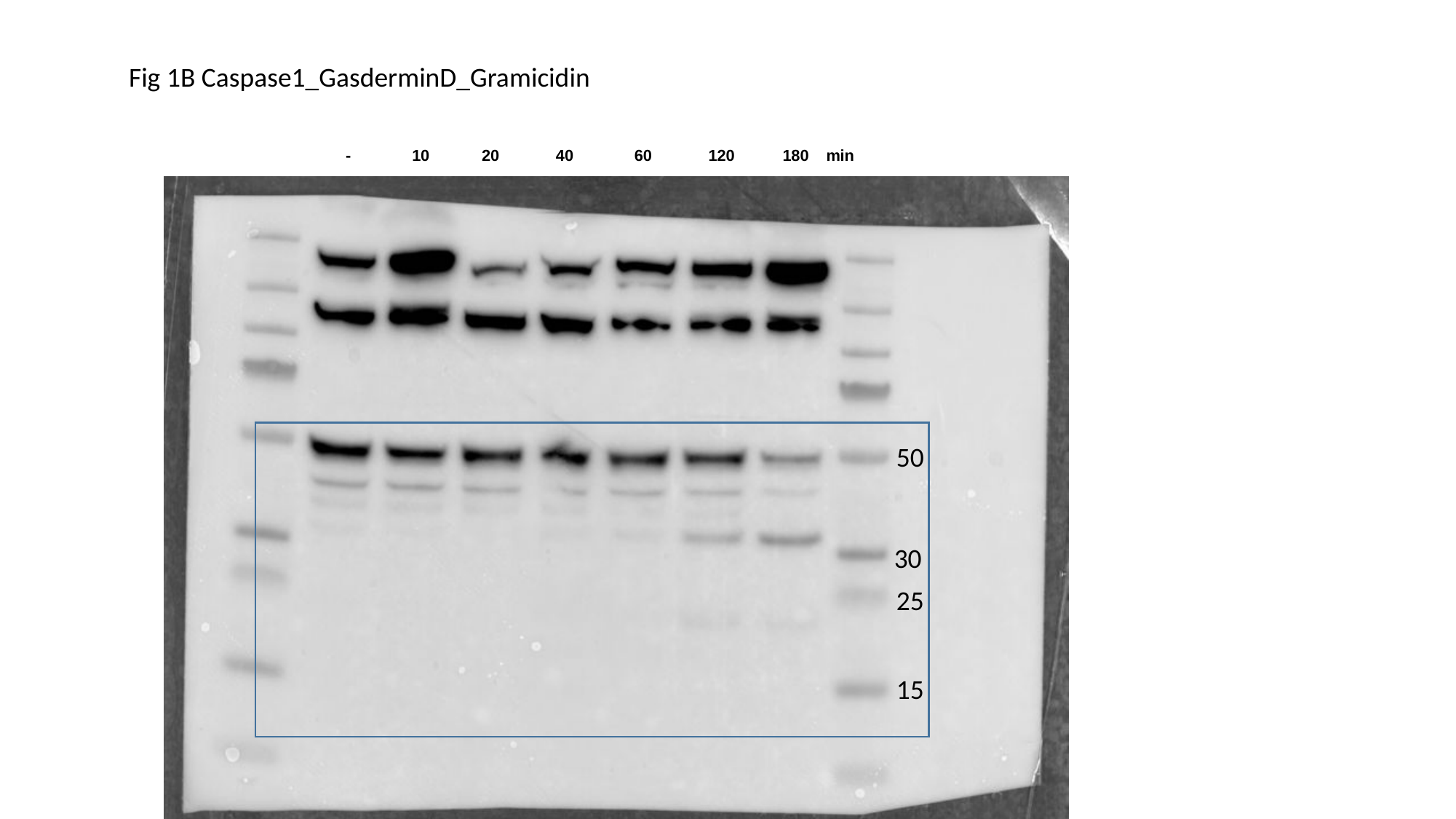

Fig 1B Caspase1_GasderminD_Gramicidin
- 10 20 40 60 120 180 min
50
30
25
15

## Slide 4
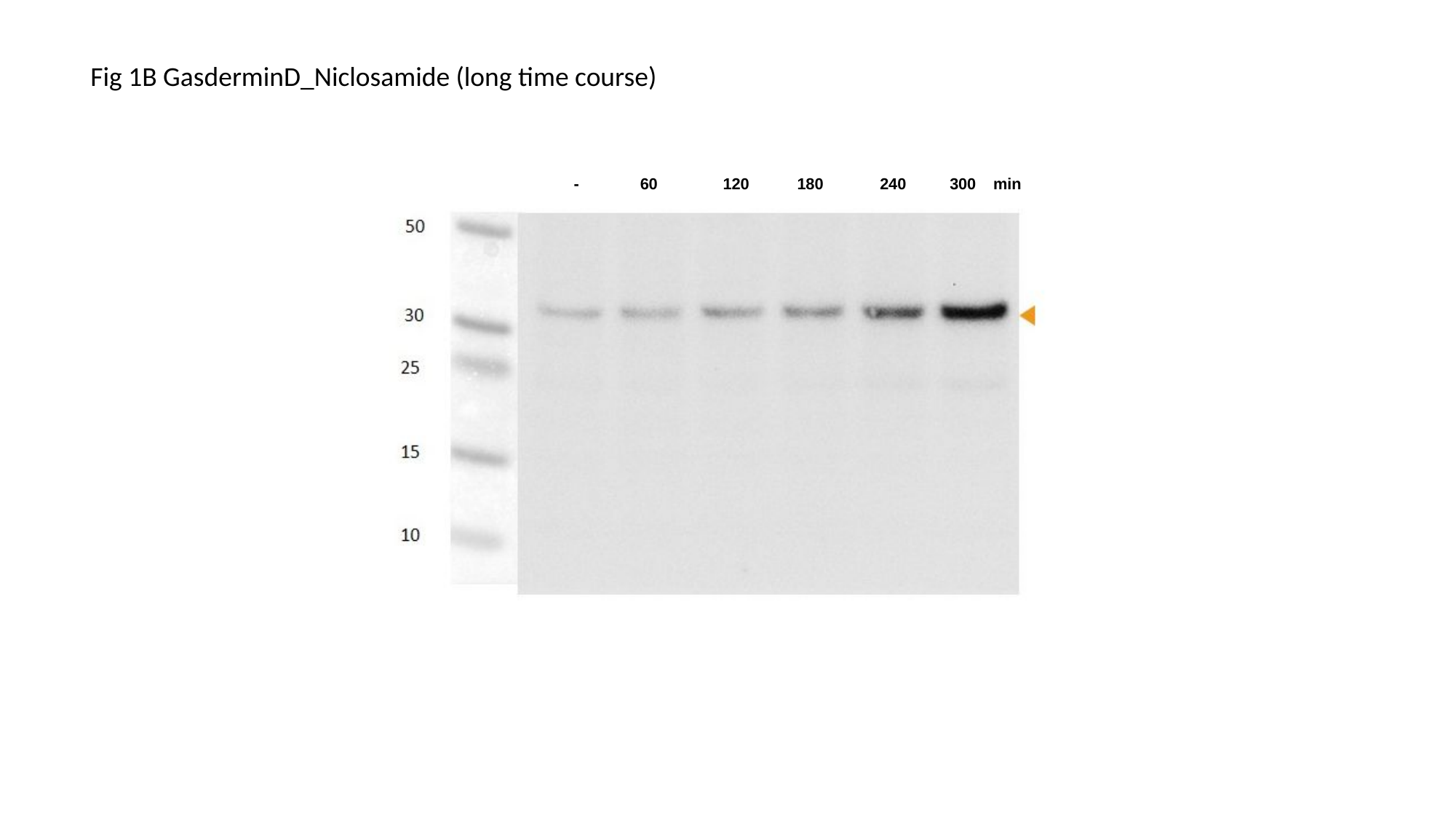

Fig 1B GasderminD_Niclosamide (long time course)
- 60 120 180 240 300 min

## Slide 5
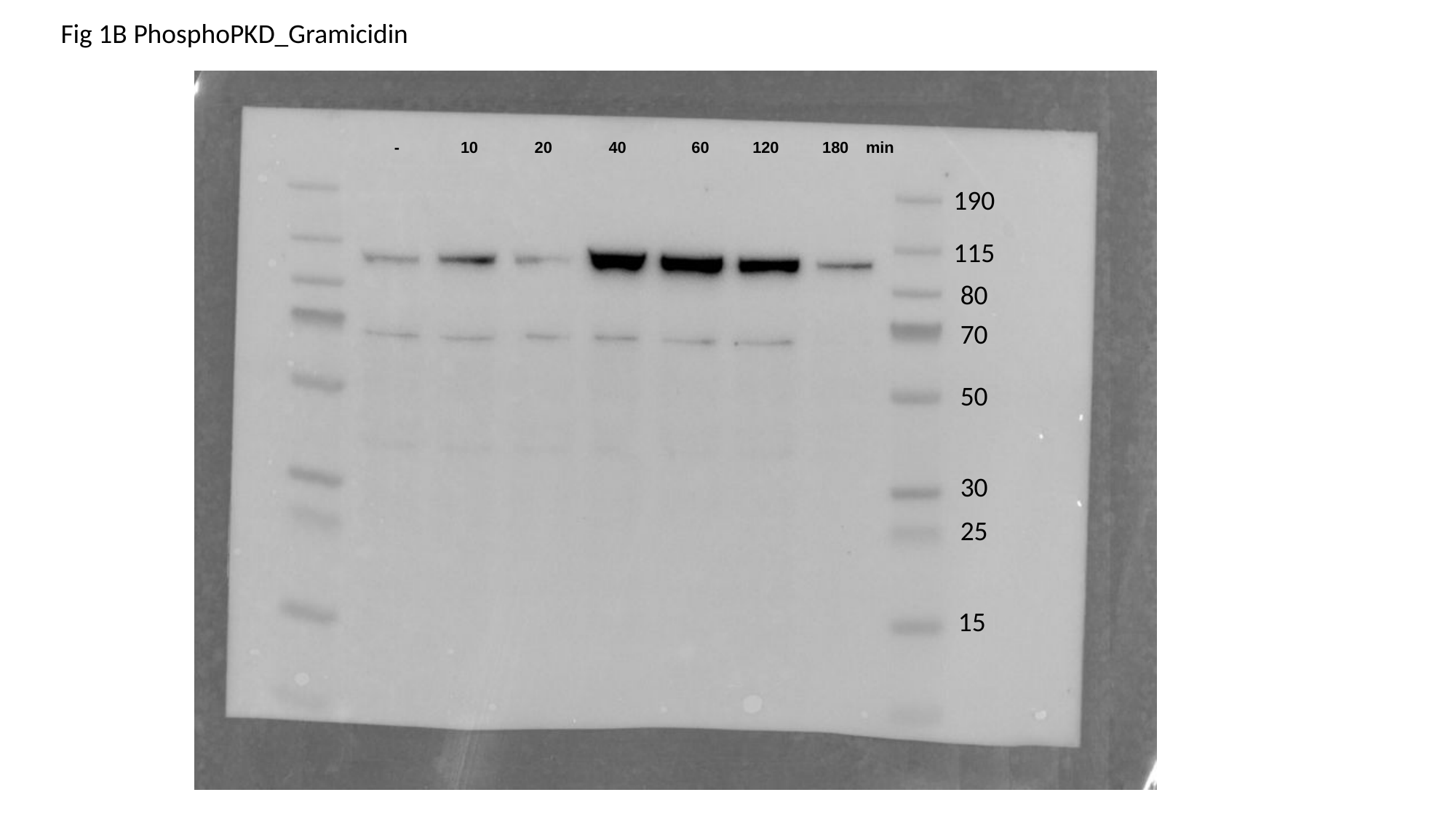

Fig 1B PhosphoPKD_Gramicidin
- 10 20 40 60 120 180 min
190
115
80
70
50
30
25
15

## Slide 6
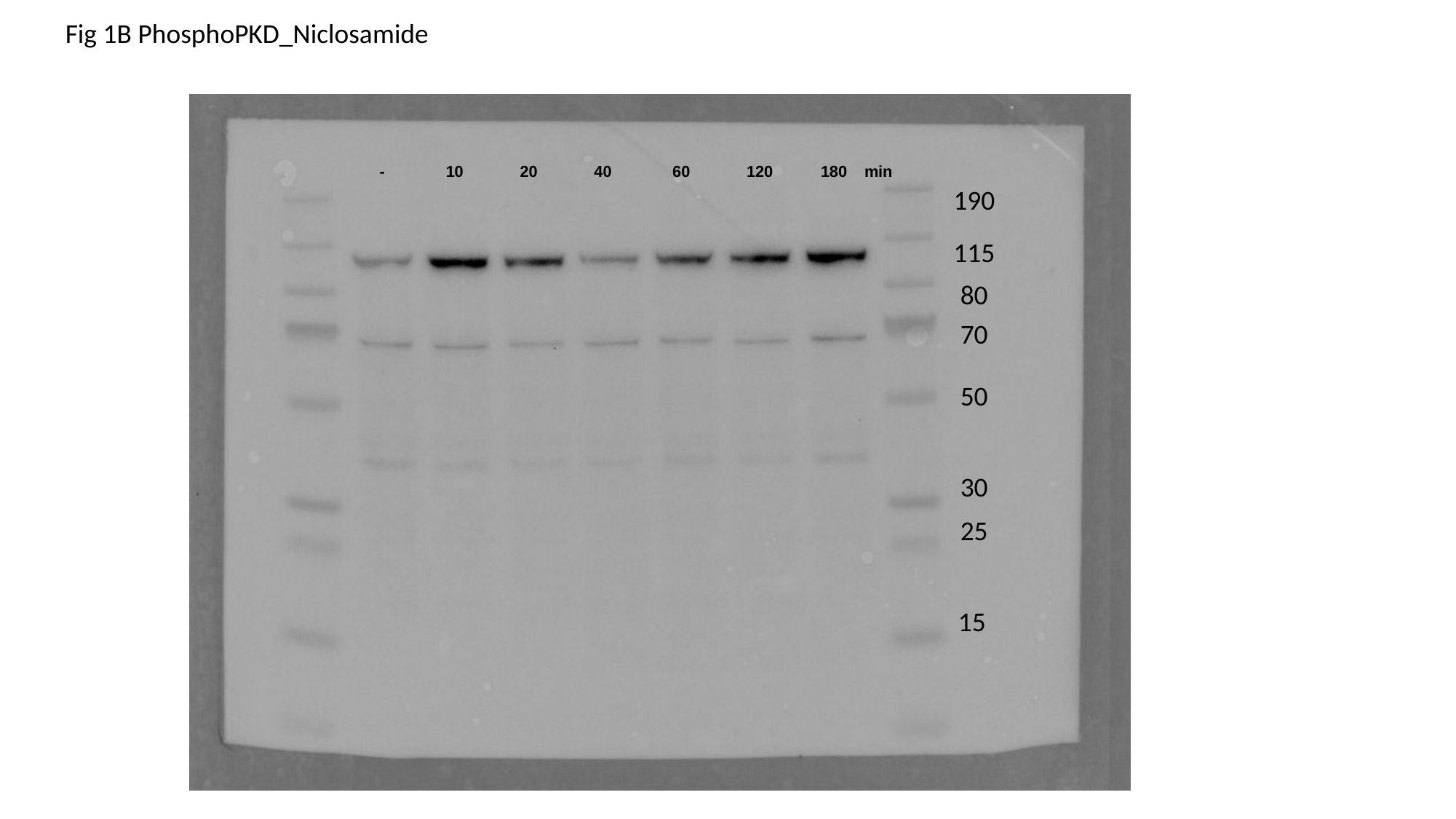

Fig 1B PhosphoPKD_Niclosamide
- 10 20 40 60 120 180 min
190
115
80
70
50
30
25
15

## Slide 7
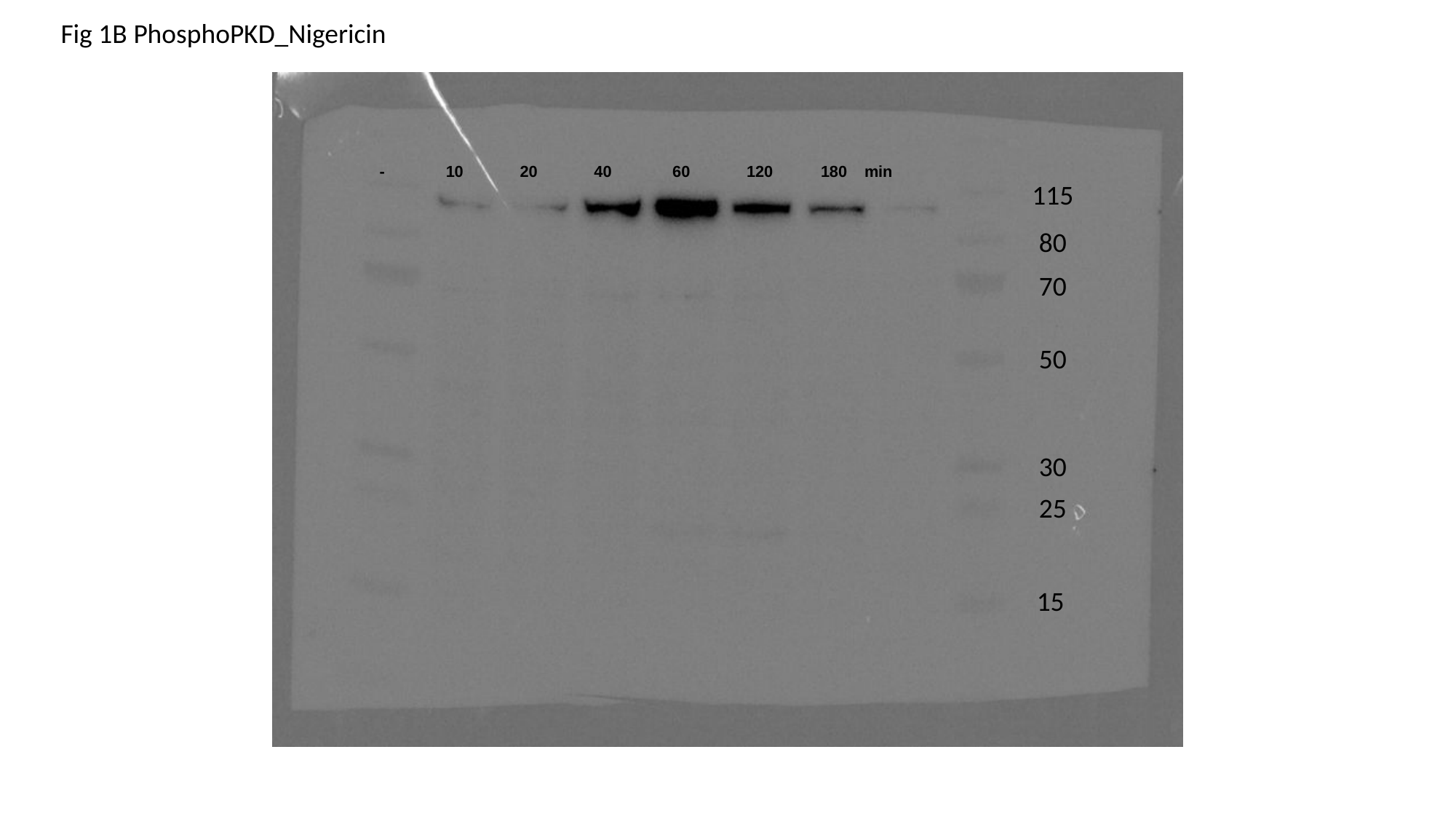

Fig 1B PhosphoPKD_Nigericin
- 10 20 40 60 120 180 min
115
80
70
50
30
25
15

## Slide 8
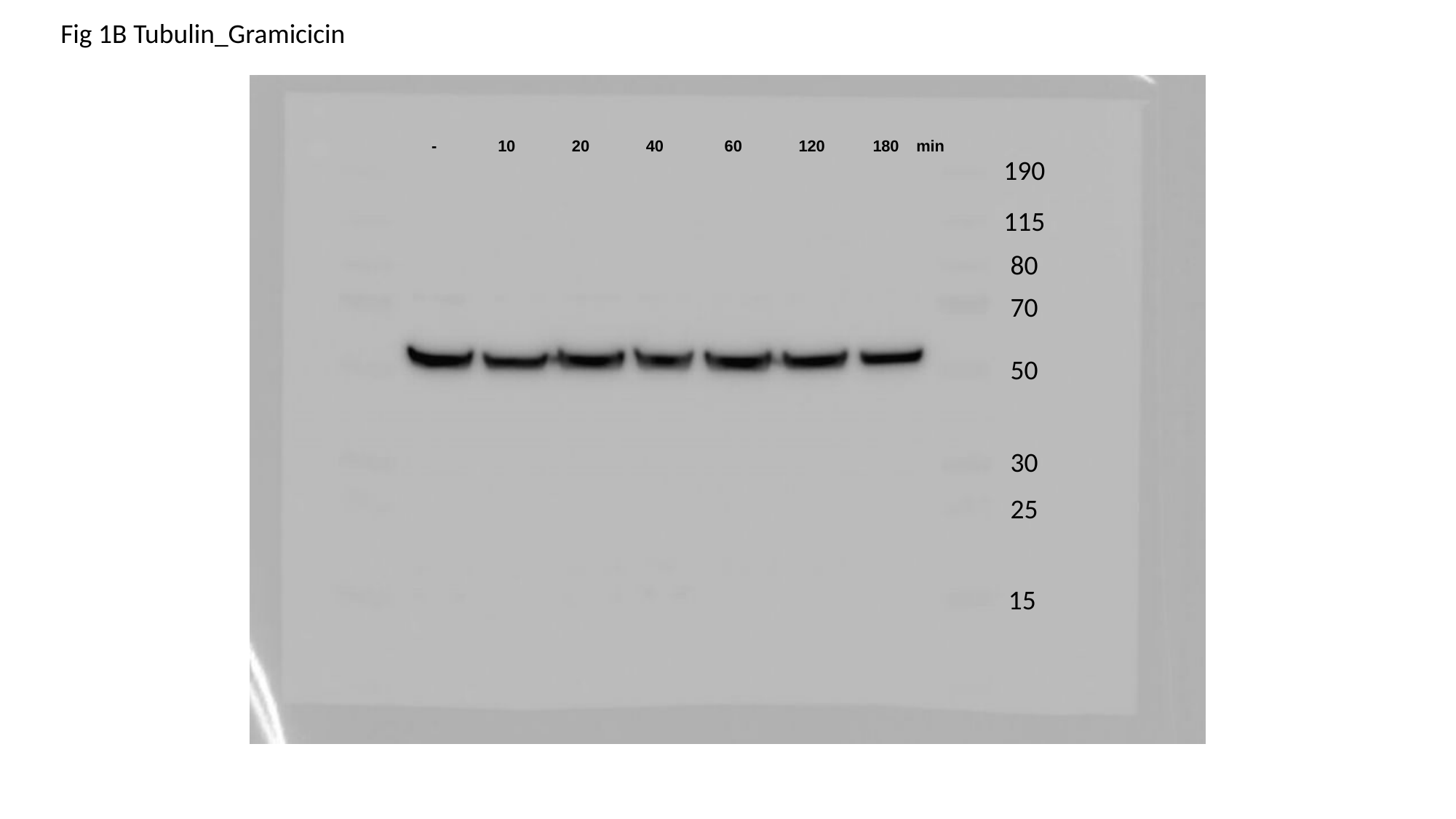

Fig 1B Tubulin_Gramicicin
- 10 20 40 60 120 180 min
190
115
80
70
50
30
25
15

## Slide 9
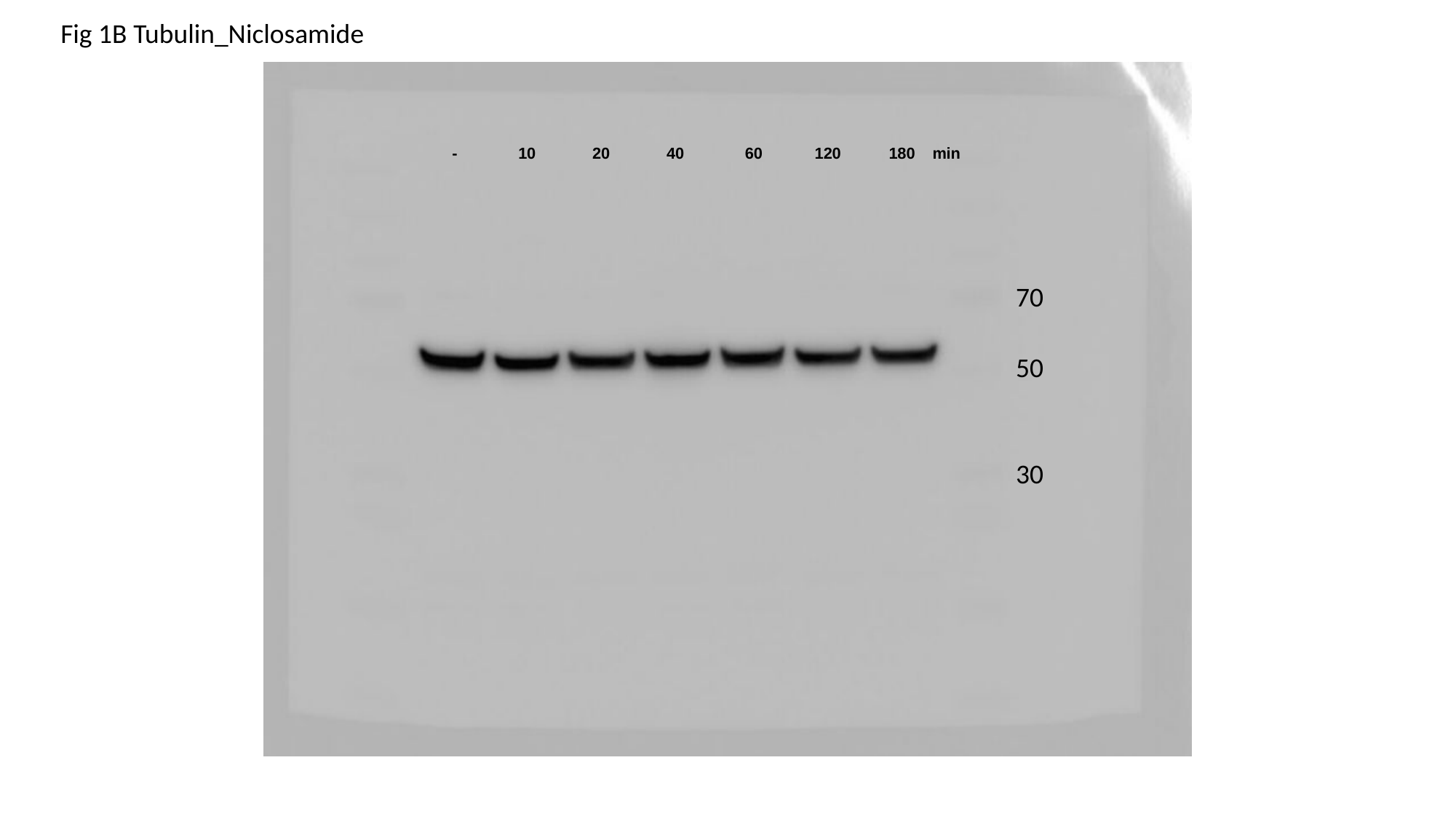

Fig 1B Tubulin_Niclosamide
- 10 20 40 60 120 180 min
70
50
30

## Slide 10
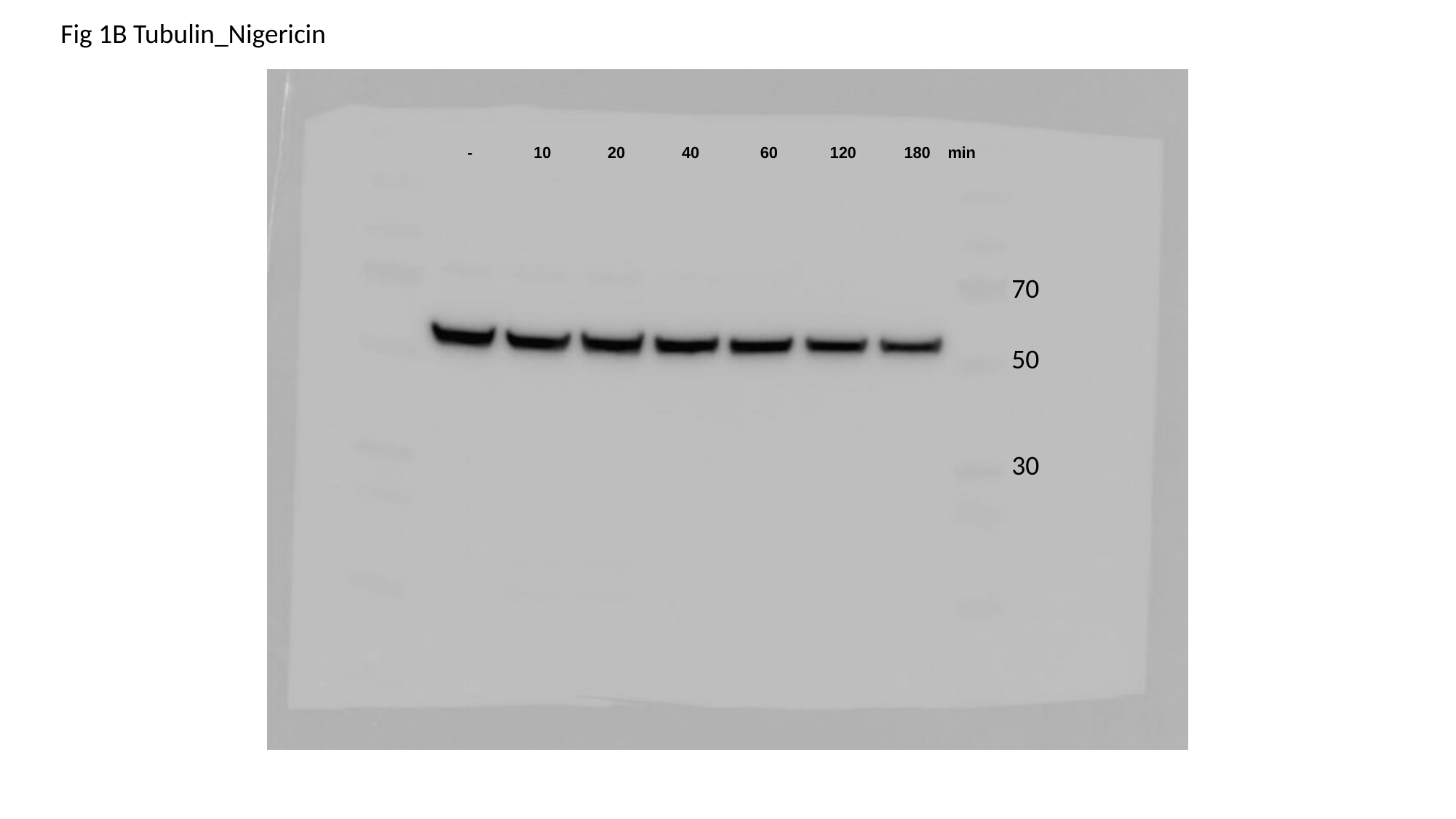

Fig 1B Tubulin_Nigericin
- 10 20 40 60 120 180 min
70
50
30

## Slide 11
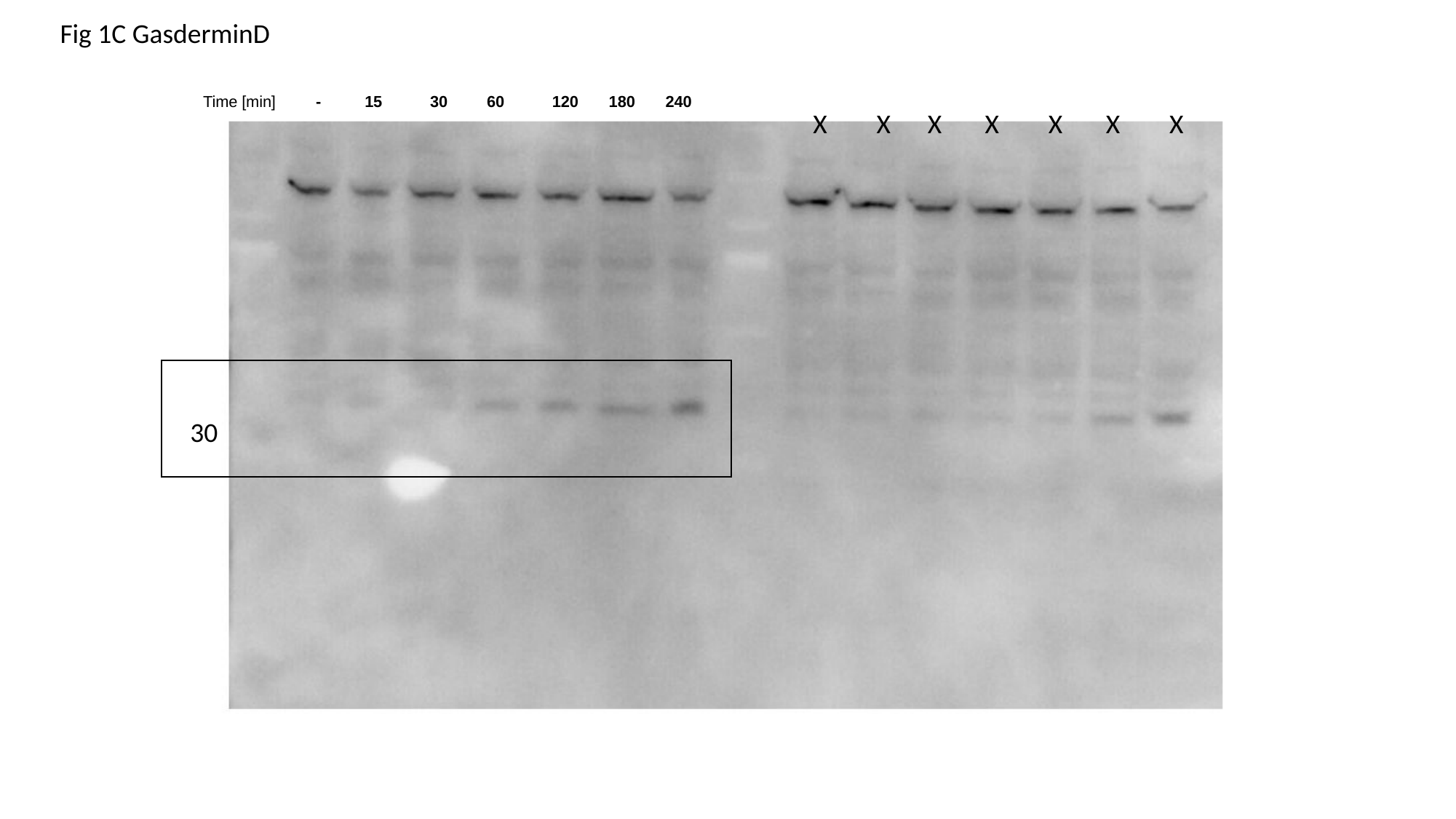

Fig 1C GasderminD
Time [min]
- 15 30 60 120 180 240
X X X X X X X
30

## Slide 12
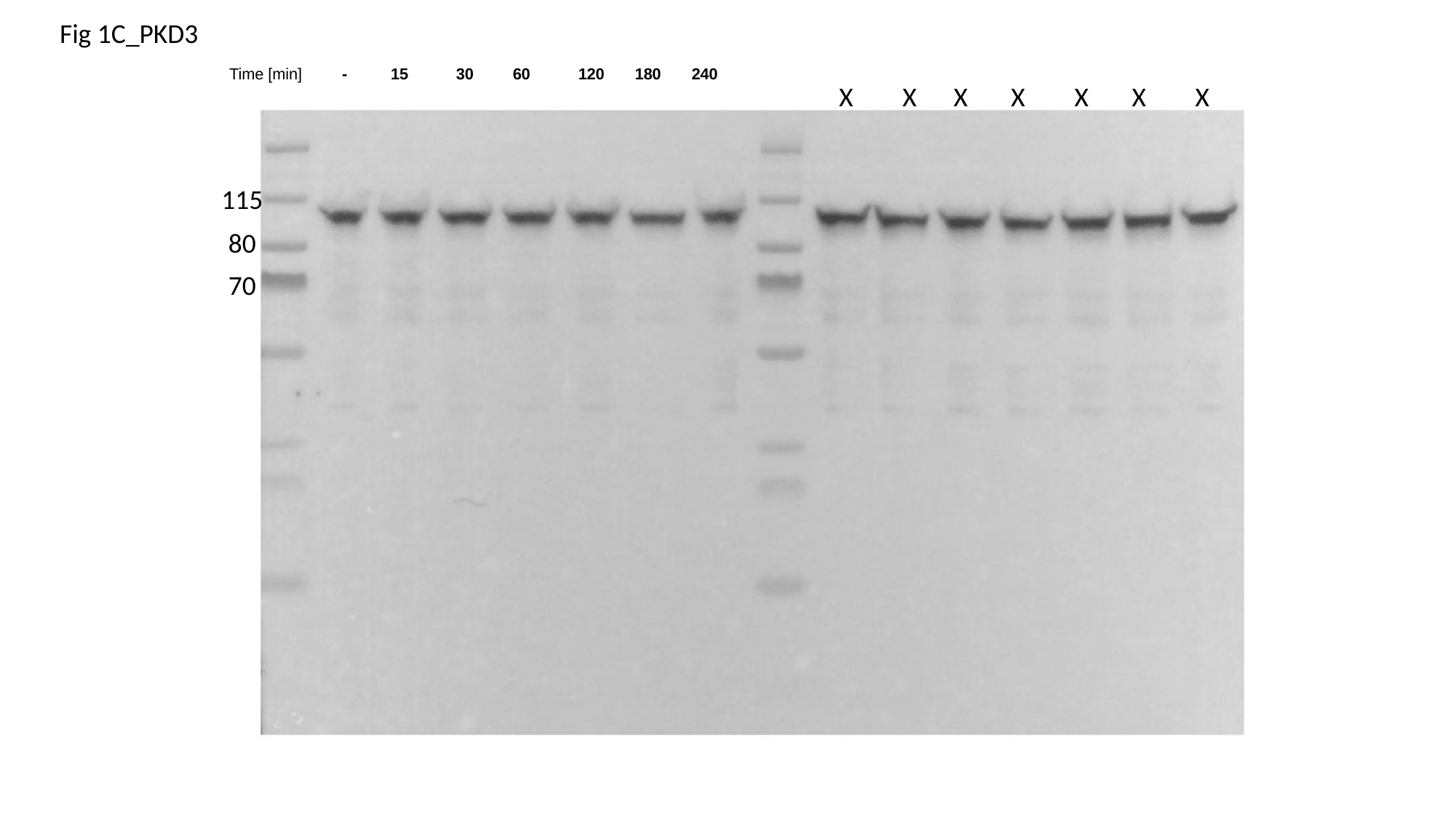

Fig 1C_PKD3
Time [min]
- 15 30 60 120 180 240
X X X X X X X
115
80
70

## Slide 13
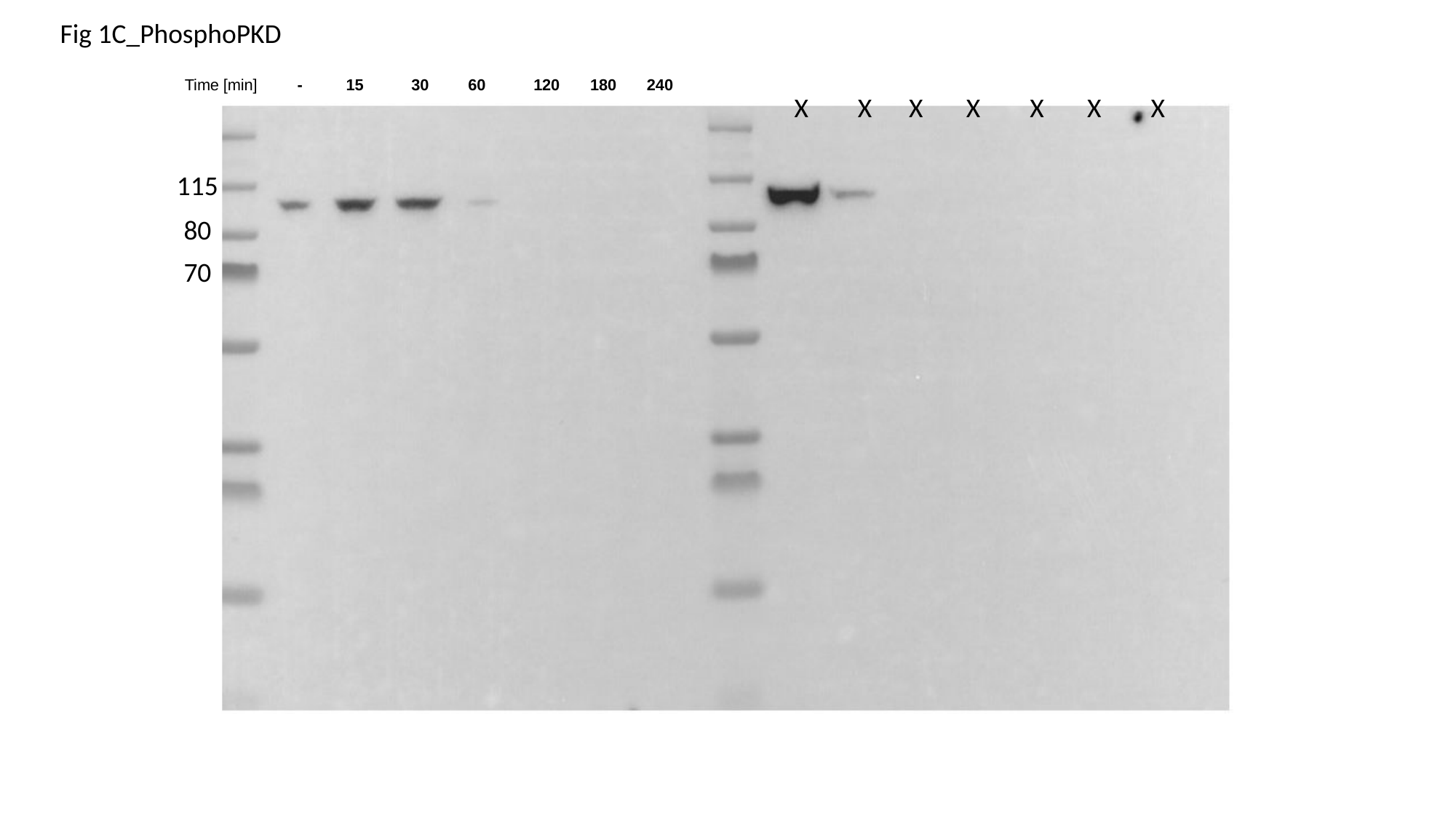

Fig 1C_PhosphoPKD
Time [min]
- 15 30 60 120 180 240
X X X X X X X
115
80
70

## Slide 14
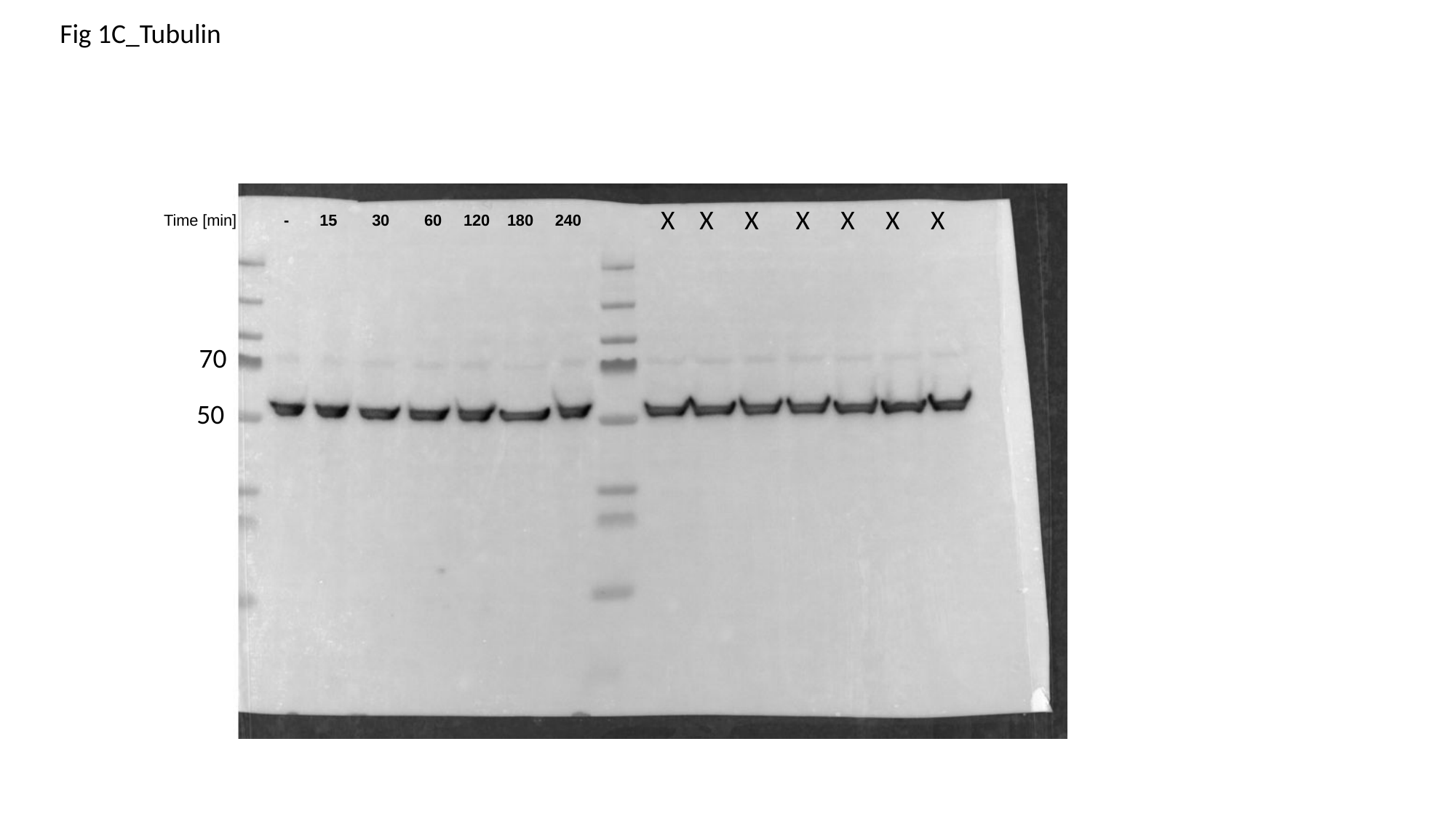

Fig 1C_Tubulin
X X X X X X X
Time [min]
- 15 30 60 120 180 240
70
50

## Slide 15
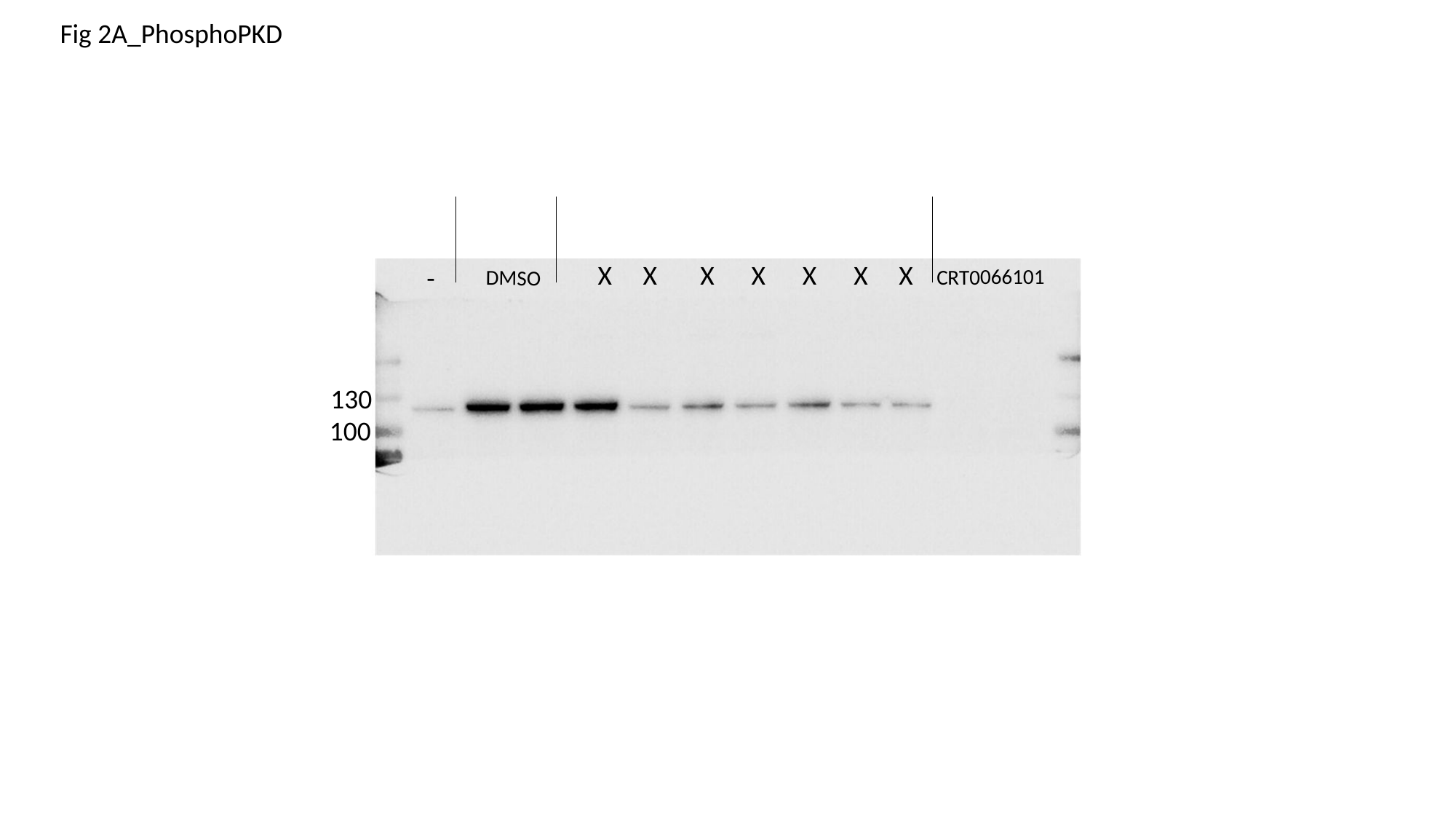

Fig 2A_PhosphoPKD
X X X X X X X
-
CRT0066101
DMSO
130
100

## Slide 16
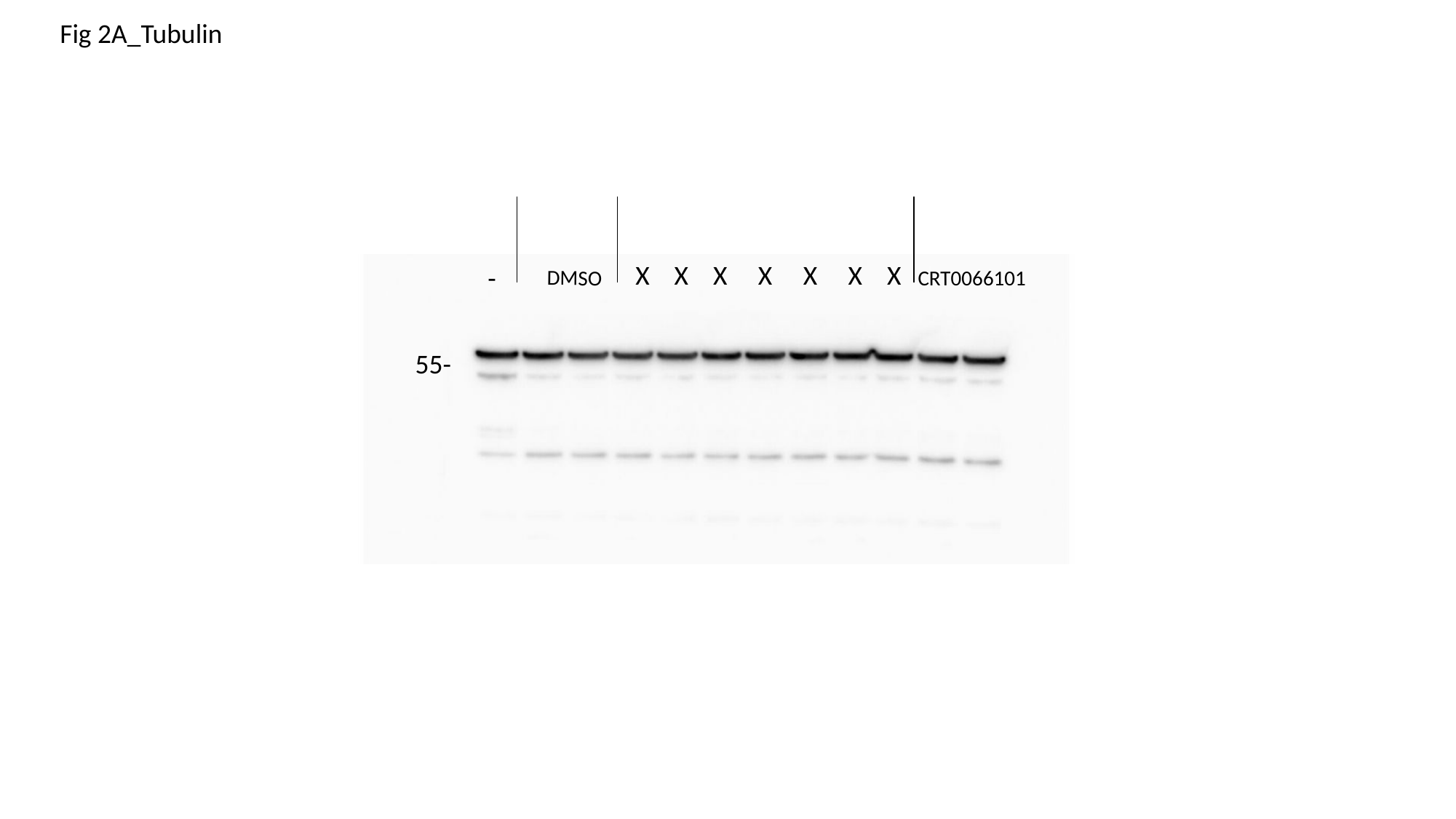

Fig 2A_Tubulin
X X X X X X X
-
DMSO
CRT0066101
55-

## Slide 17
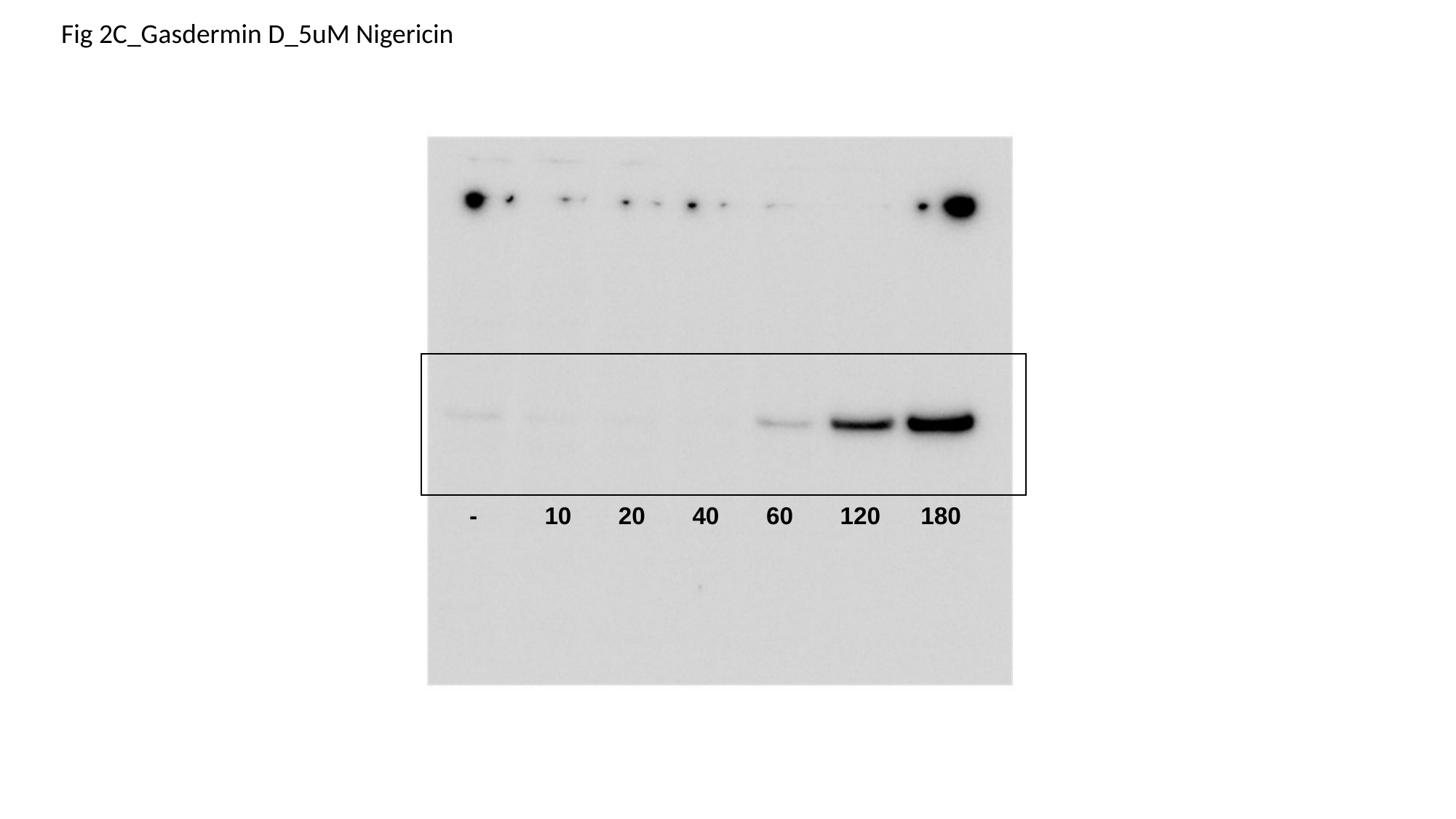

Fig 2C_Gasdermin D_5uM Nigericin
- 10 20 40 60 120 180

## Slide 18
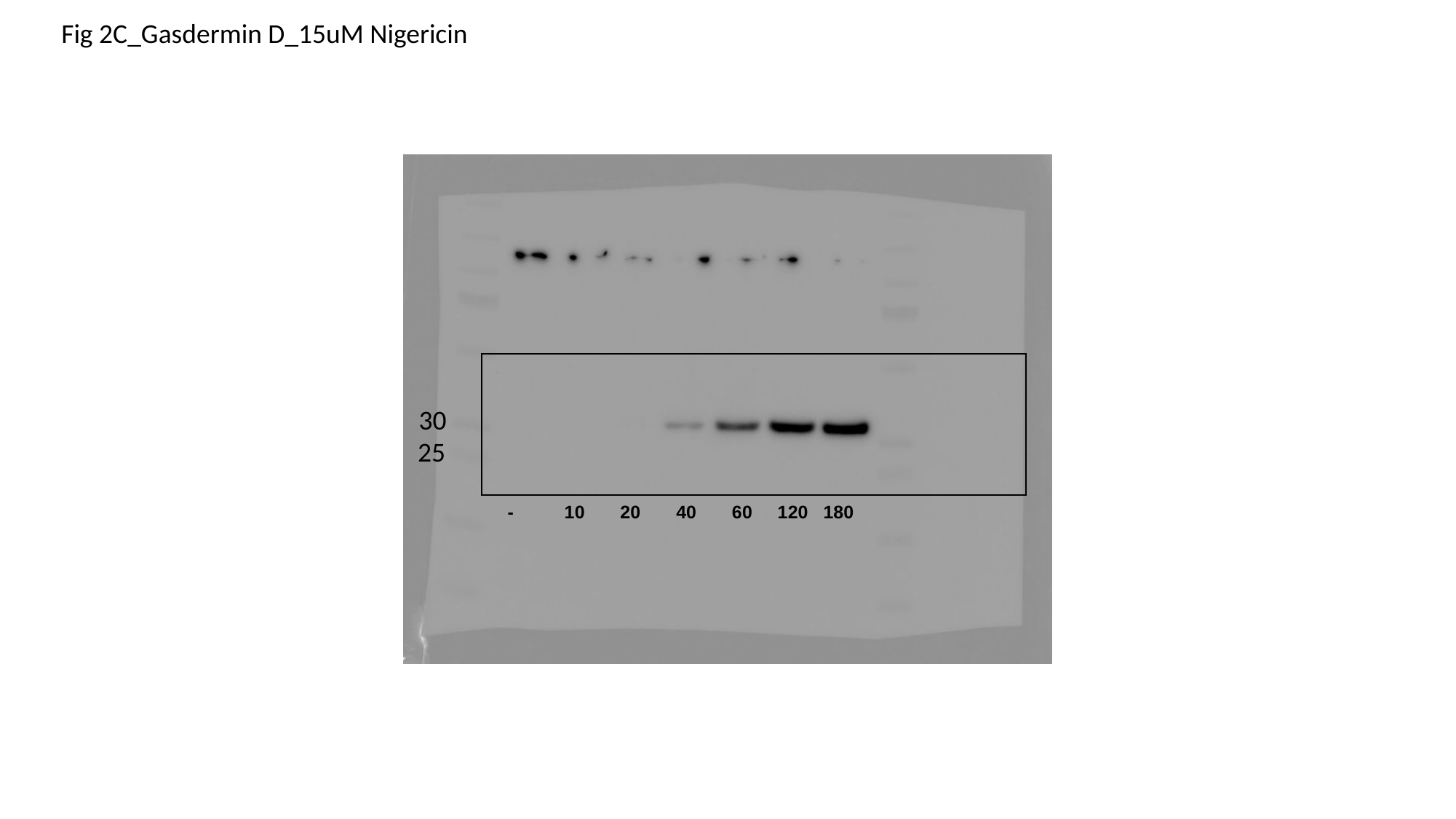

Fig 2C_Gasdermin D_15uM Nigericin
30
25
- 10 20 40 60 120 180

## Slide 19
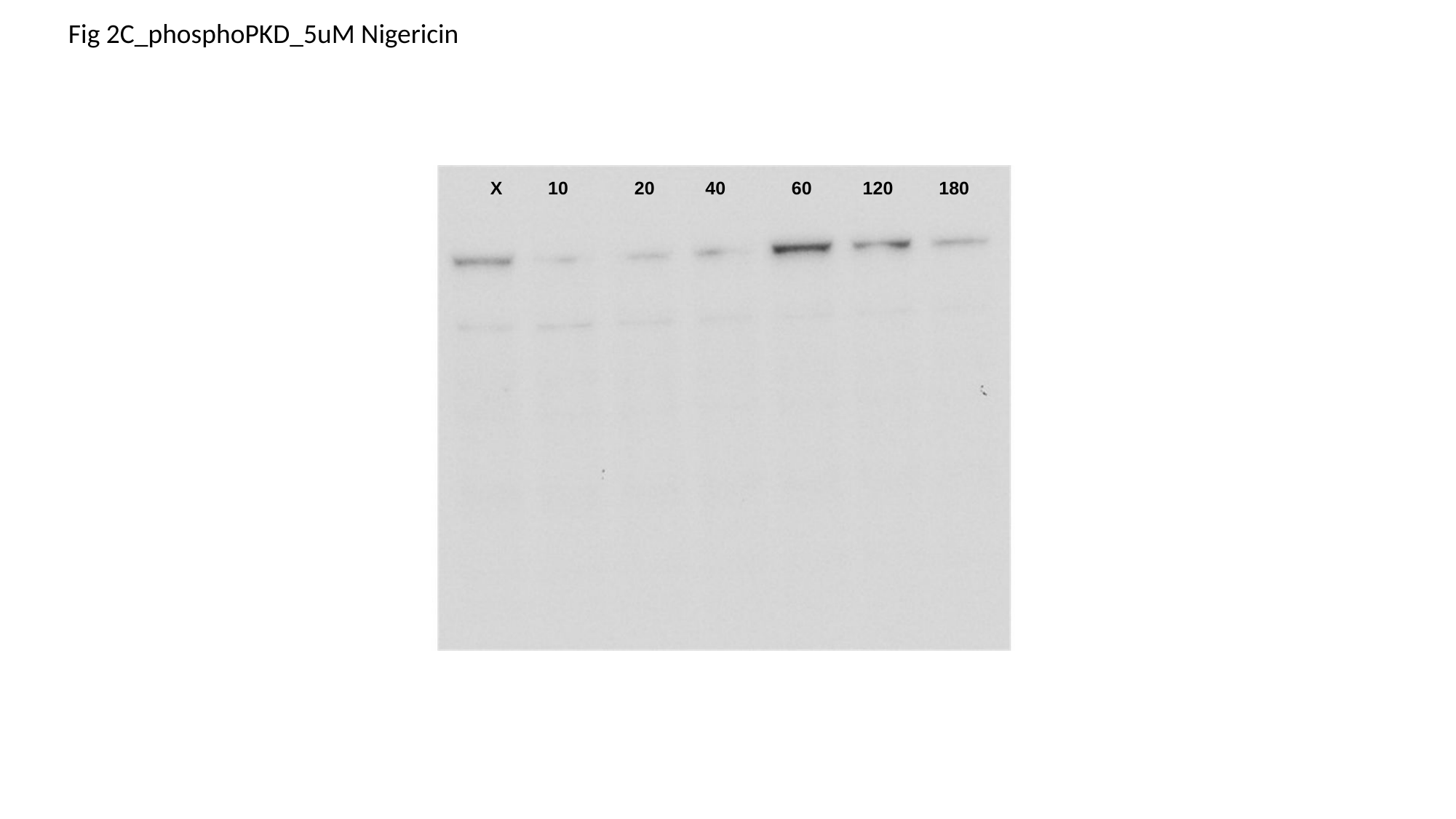

Fig 2C_phosphoPKD_5uM Nigericin
X 10 20 40 60 120 180

## Slide 20
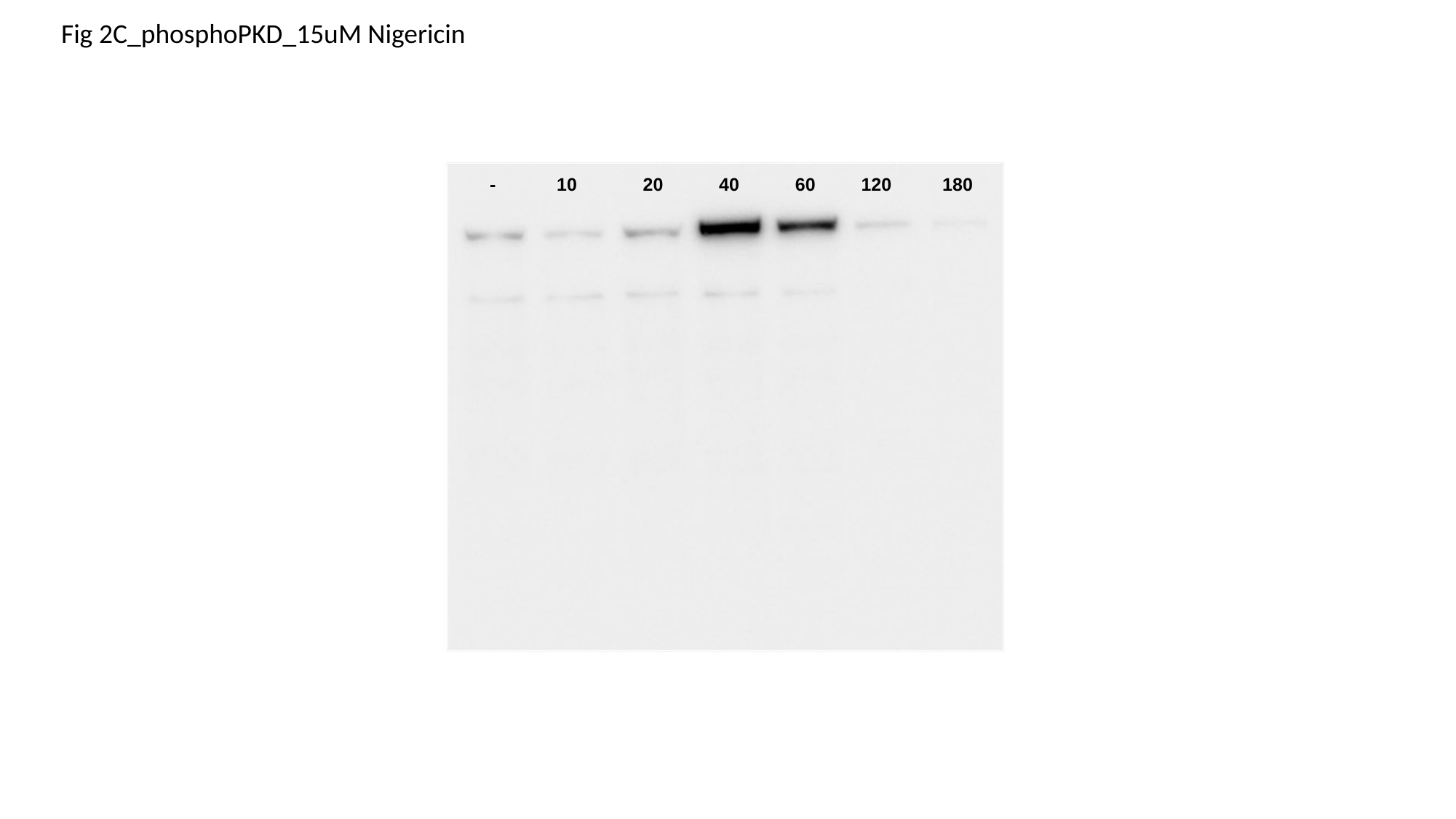

Fig 2C_phosphoPKD_15uM Nigericin
- 10 20 40 60 120 180

## Slide 21
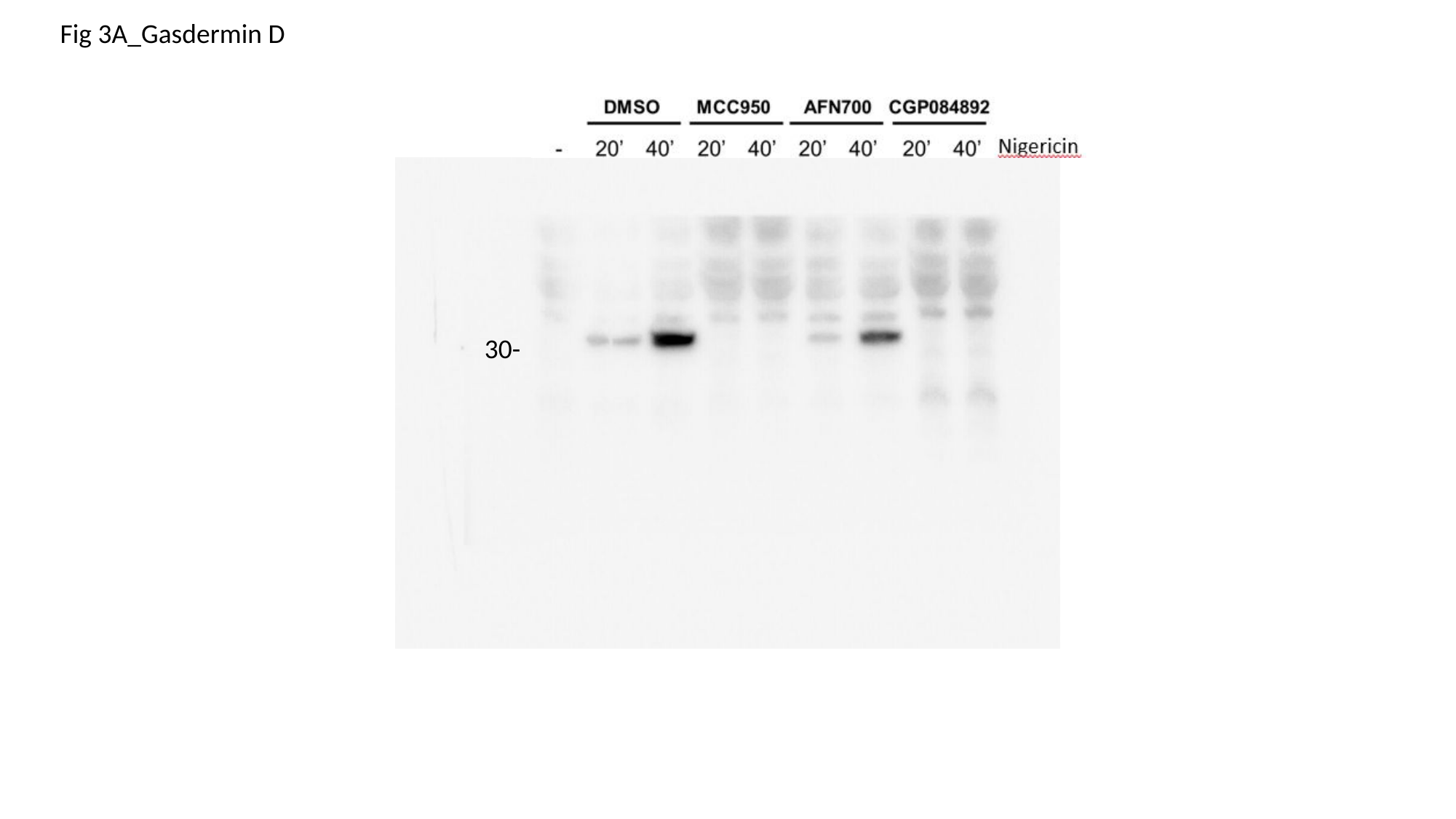

Fig 3A_Gasdermin D
30-

## Slide 22
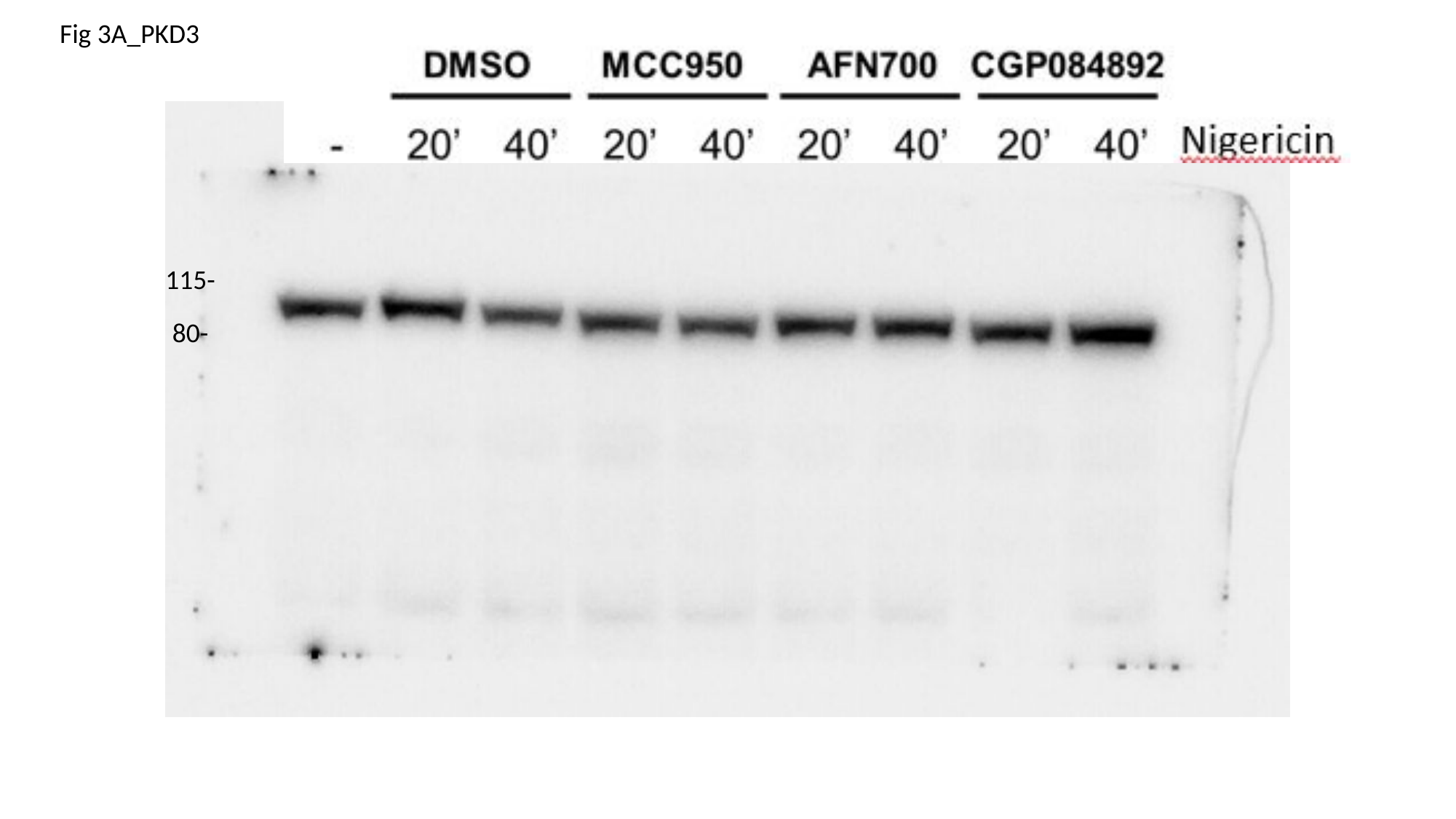

Fig 3A_PKD3
115-
80-

## Slide 23
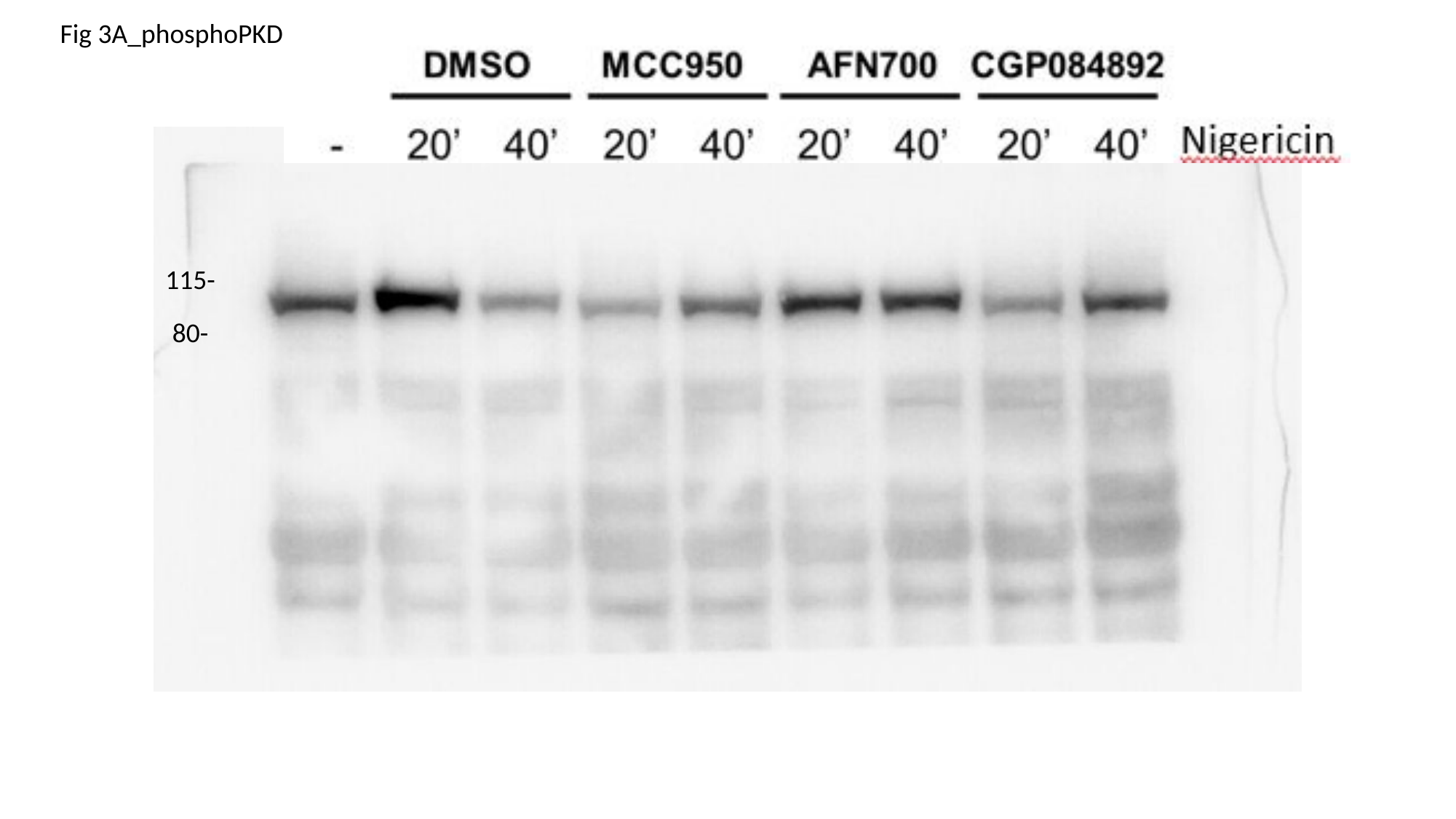

Fig 3A_phosphoPKD
115-
80-

## Slide 24
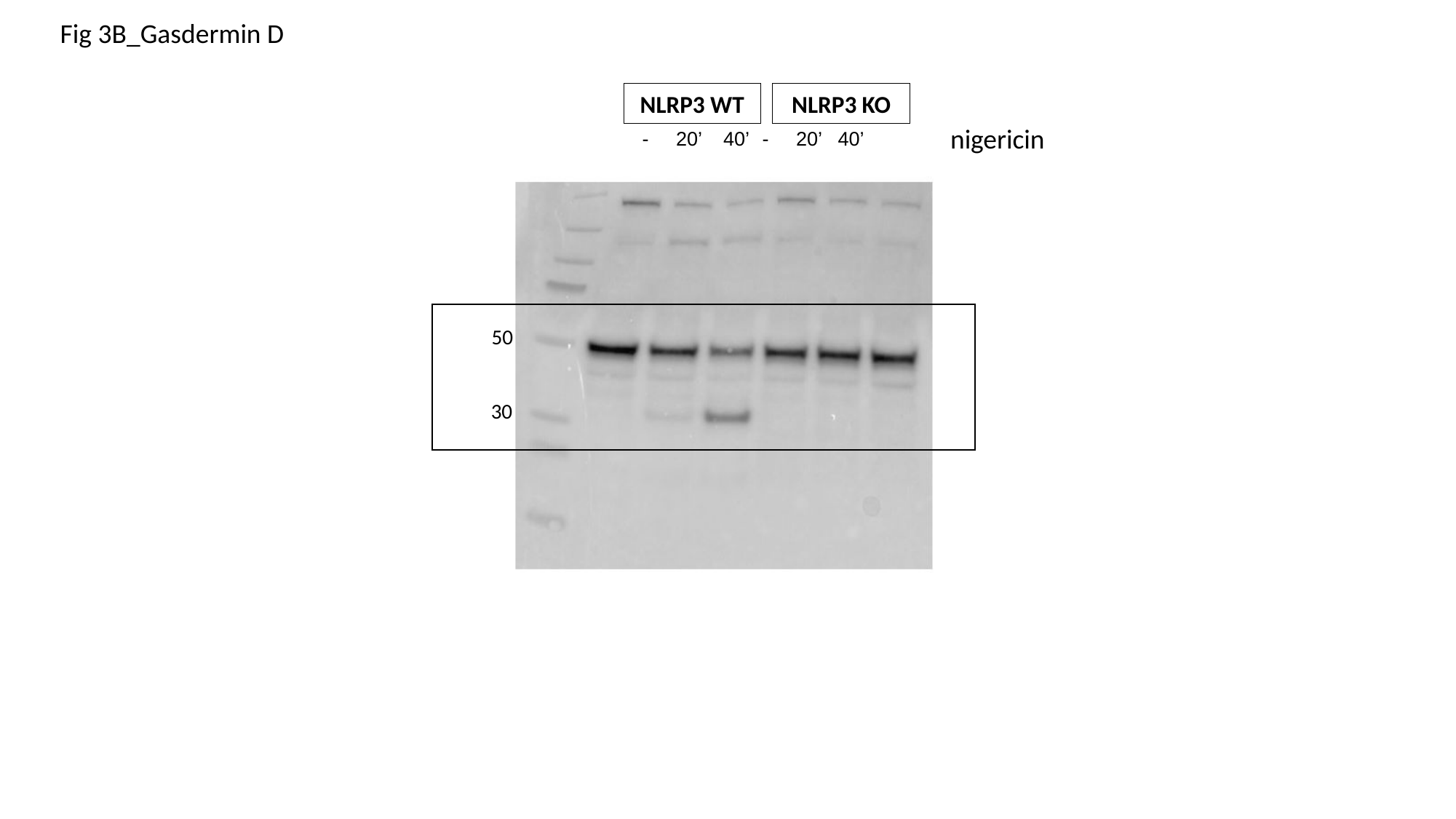

Fig 3B_Gasdermin D
NLRP3 WT
NLRP3 KO
nigericin
- 20’ 40’	 - 20’ 40’
50
30

## Slide 25
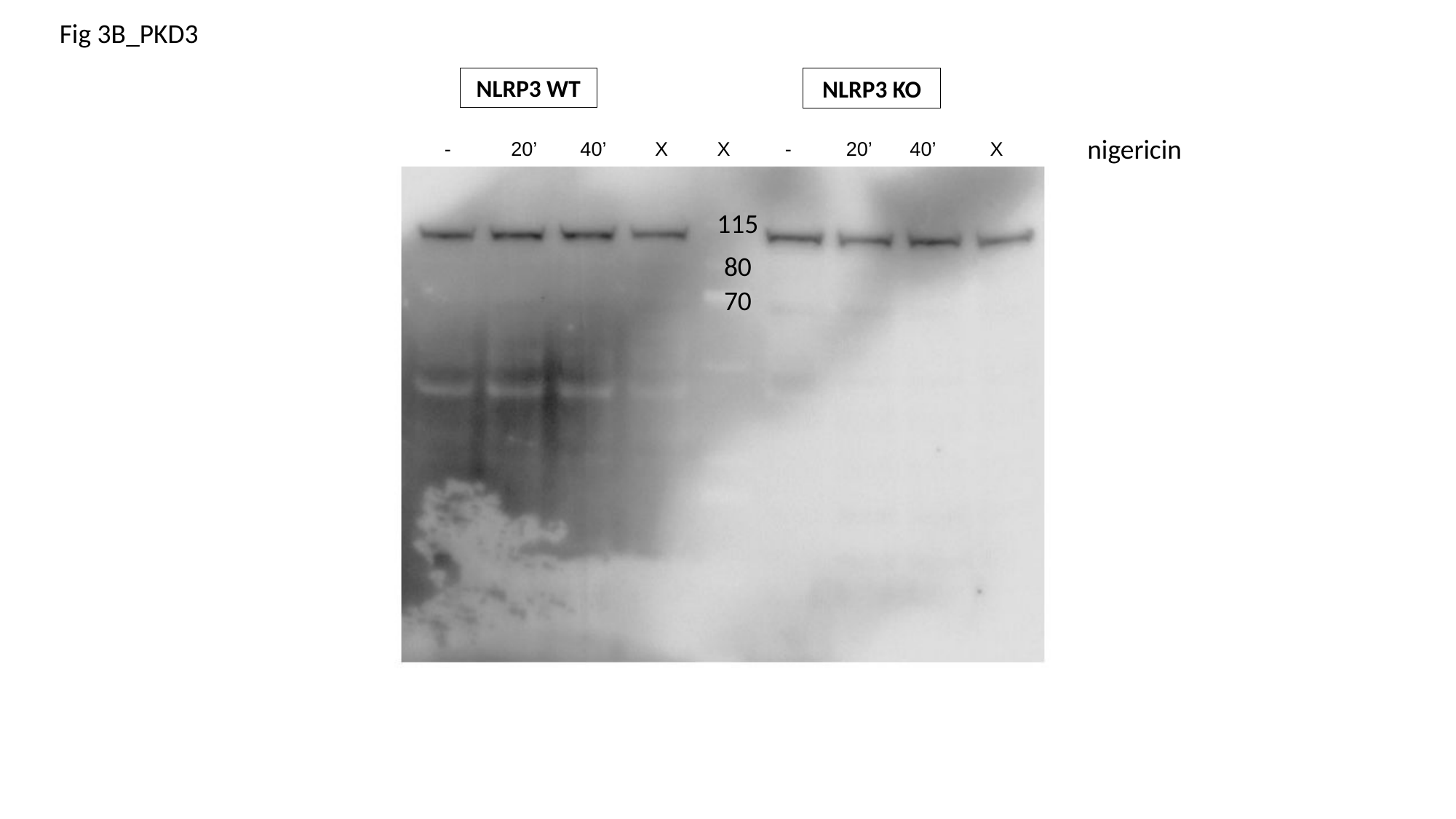

Fig 3B_PKD3
NLRP3 WT
NLRP3 KO
nigericin
- 20’ 40’ X X - 20’ 40’ X
115
80
70

## Slide 26
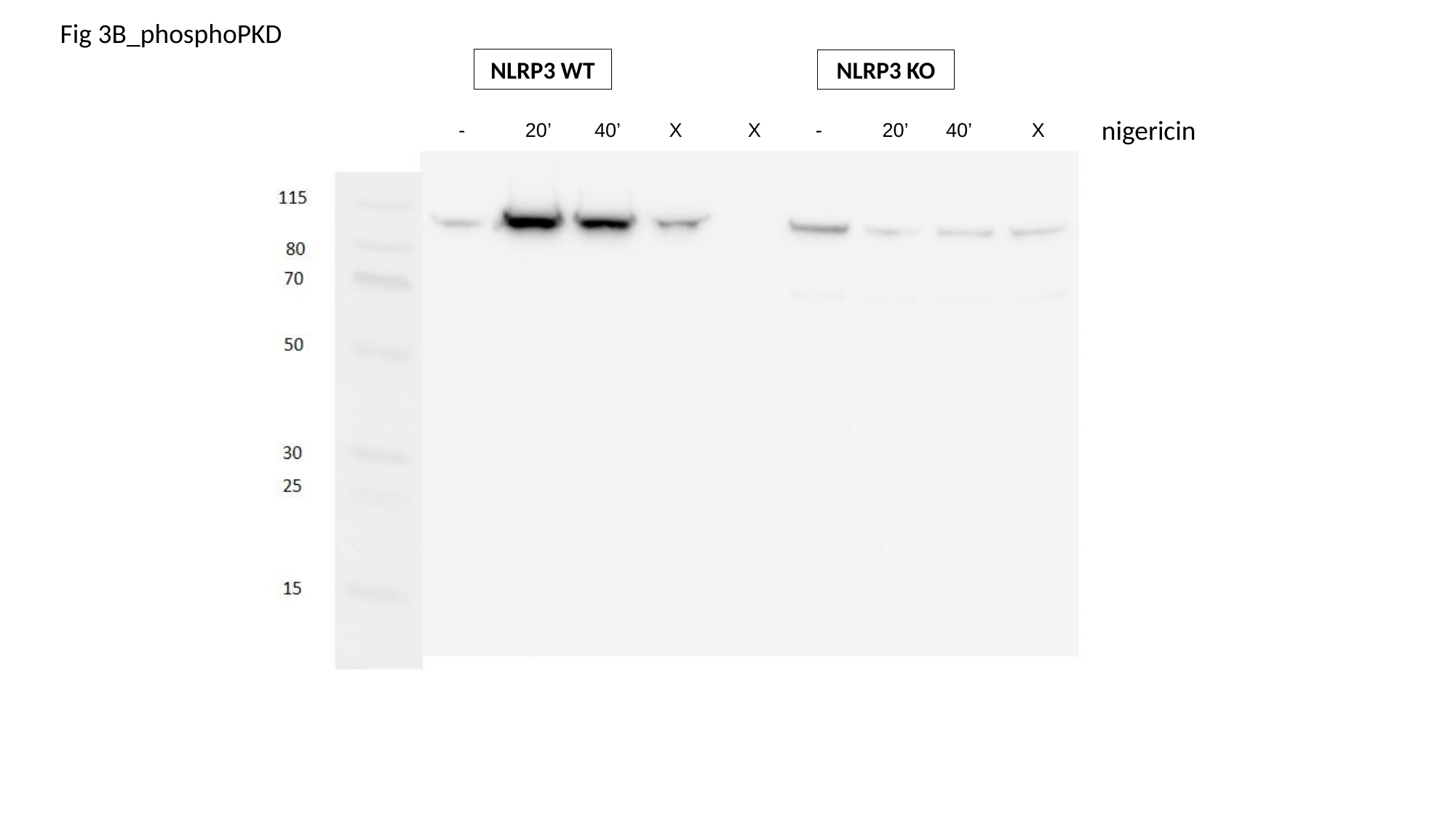

Fig 3B_phosphoPKD
NLRP3 WT
NLRP3 KO
nigericin
- 20’ 40’ X X - 20’ 40’ X

## Slide 27
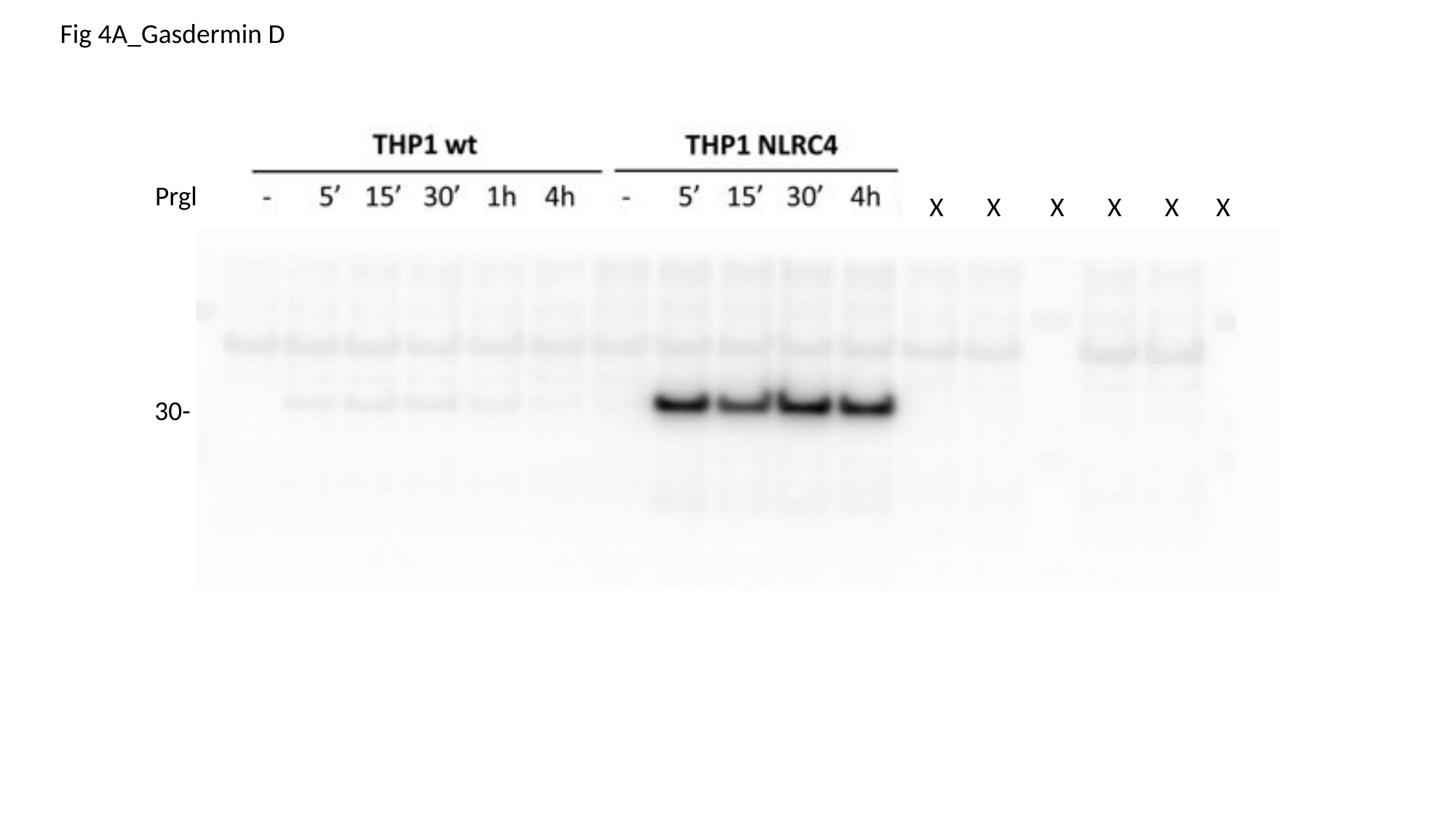

Fig 4A_Gasdermin D
Prgl
X X X X X X
30-

## Slide 28
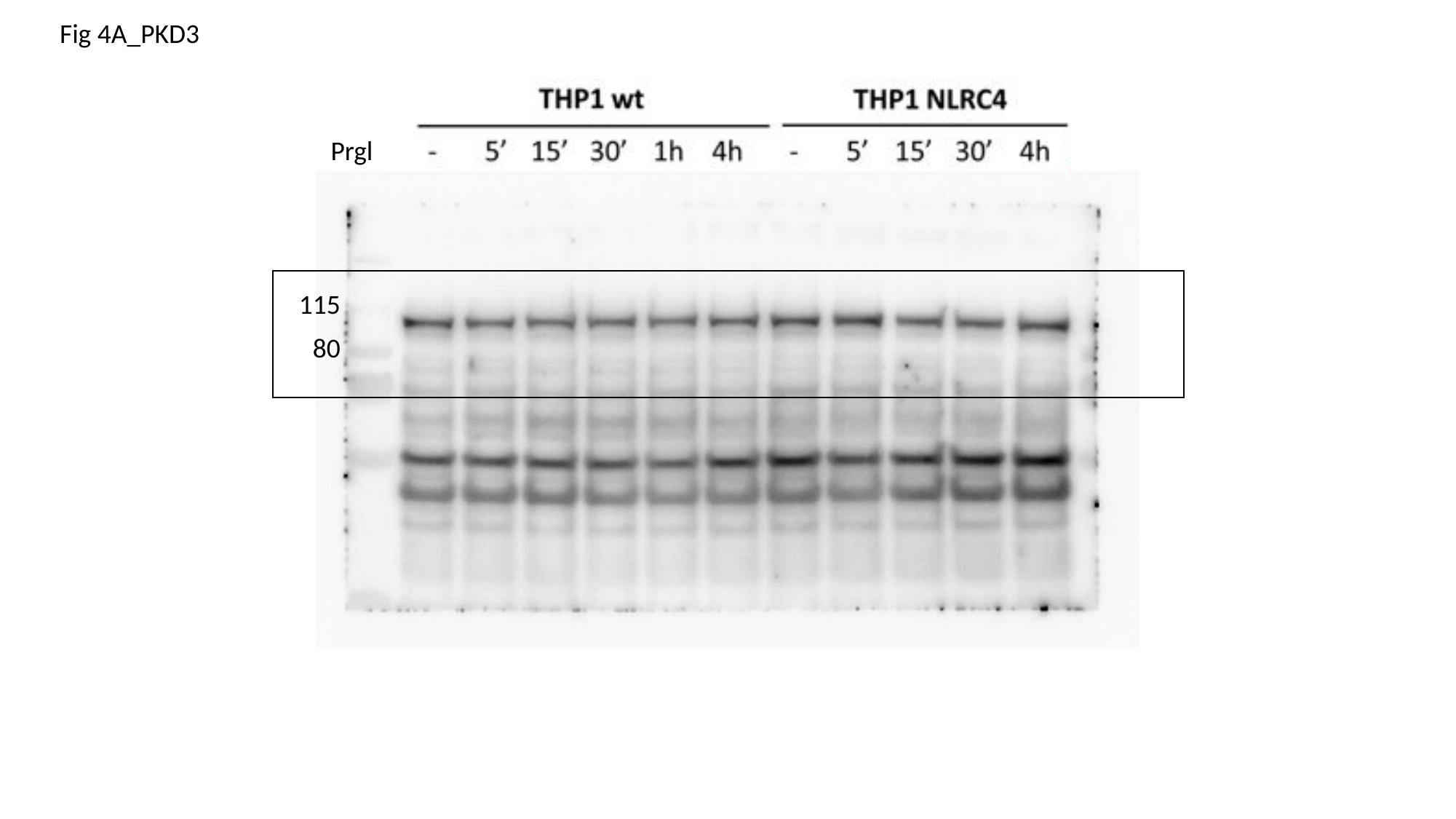

Fig 4A_PKD3
Prgl
115
80

## Slide 29
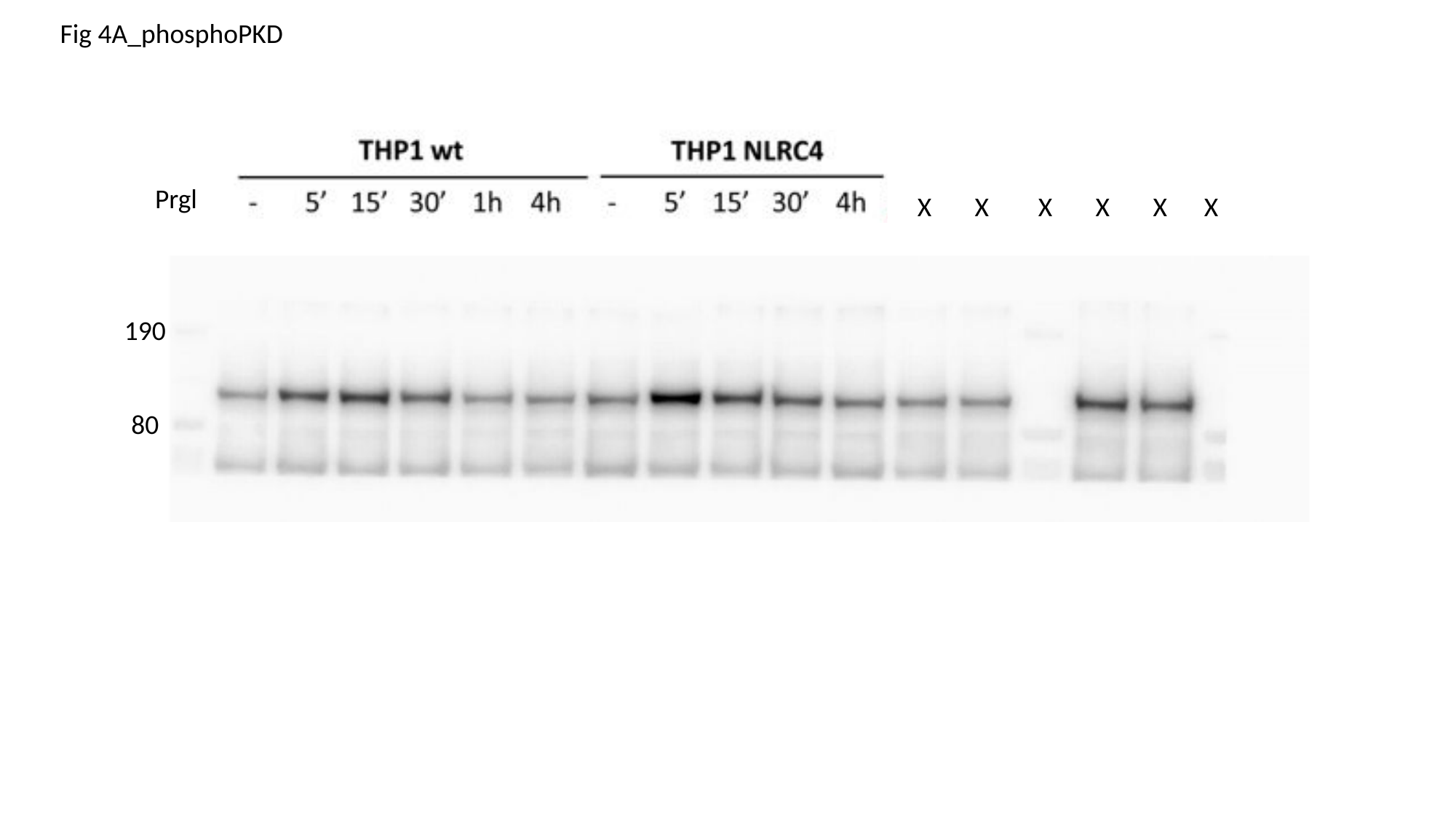

Fig 4A_phosphoPKD
Prgl
X X X X X X
190
80

## Slide 30
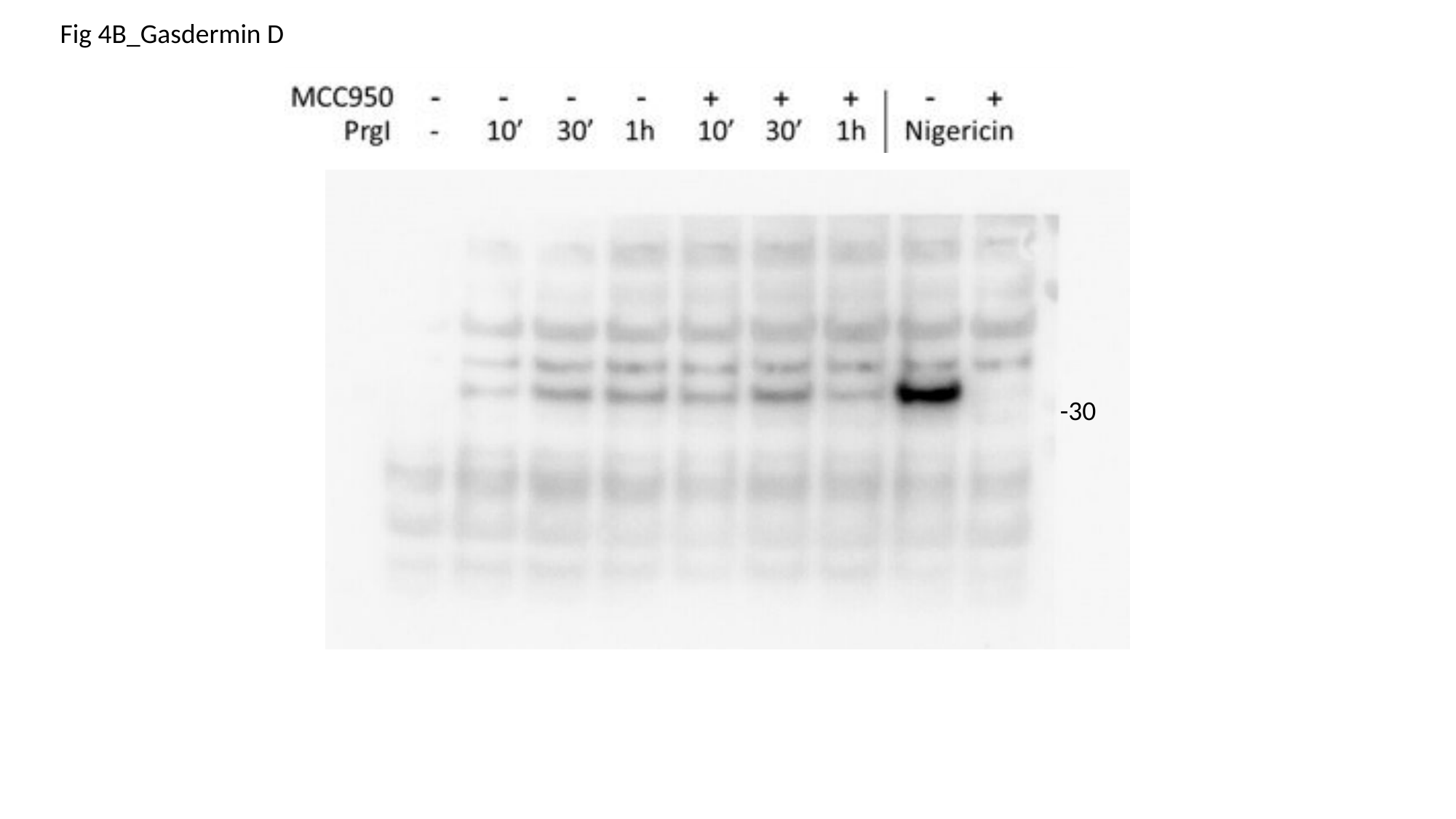

Fig 4B_Gasdermin D
-30

## Slide 31
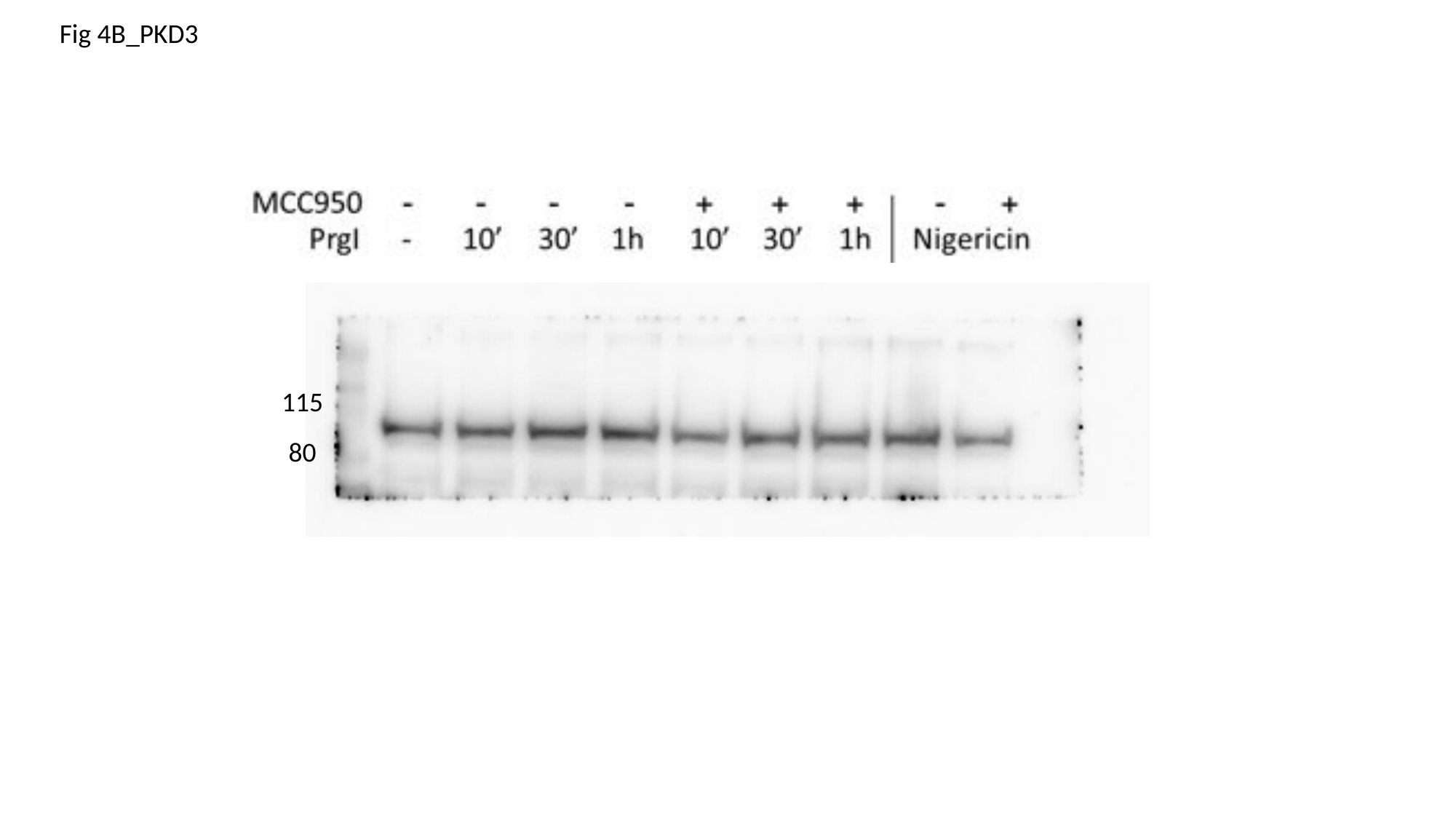

Fig 4B_PKD3
115
80

## Slide 32
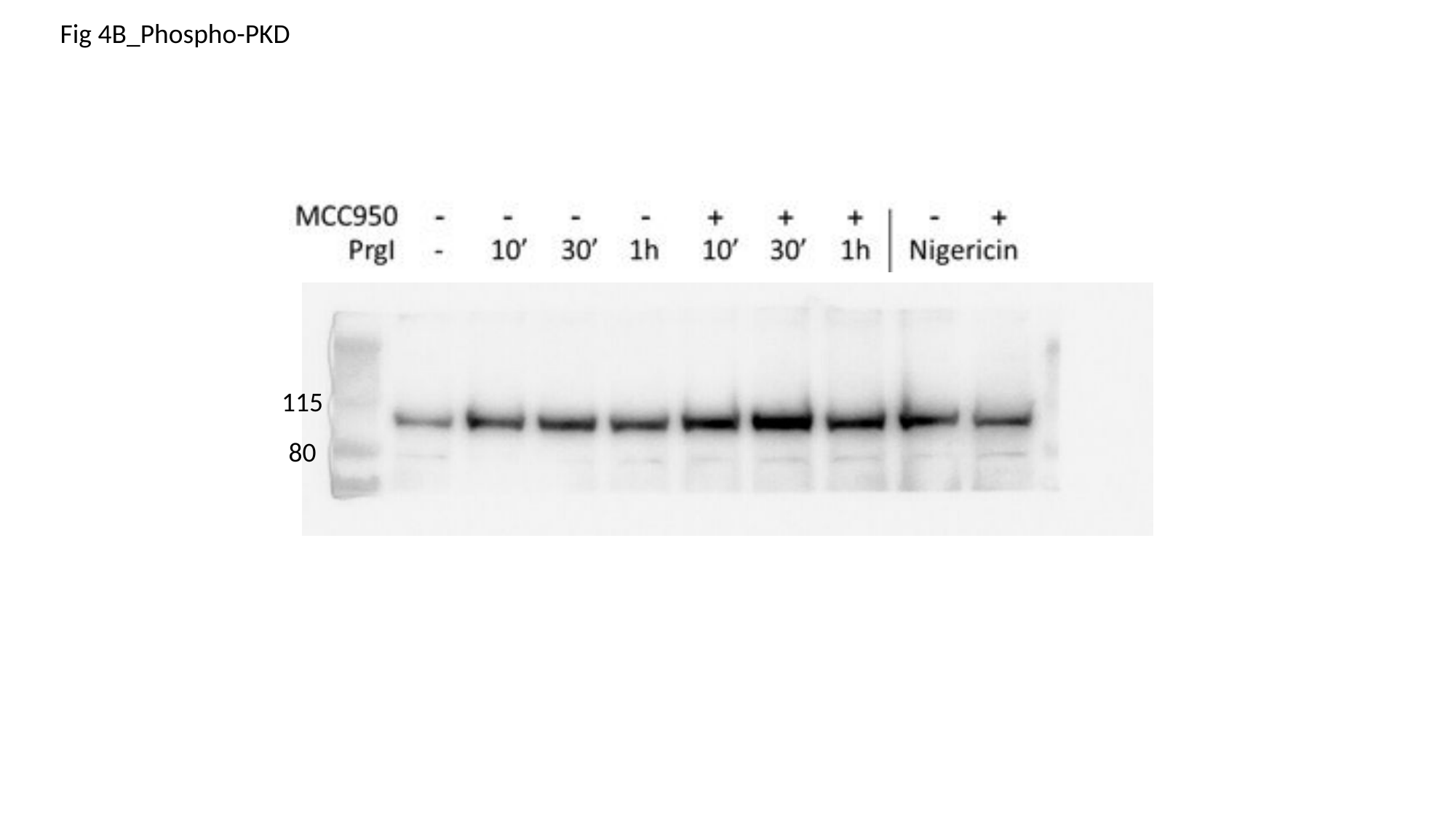

Fig 4B_Phospho-PKD
115
80

## Slide 33
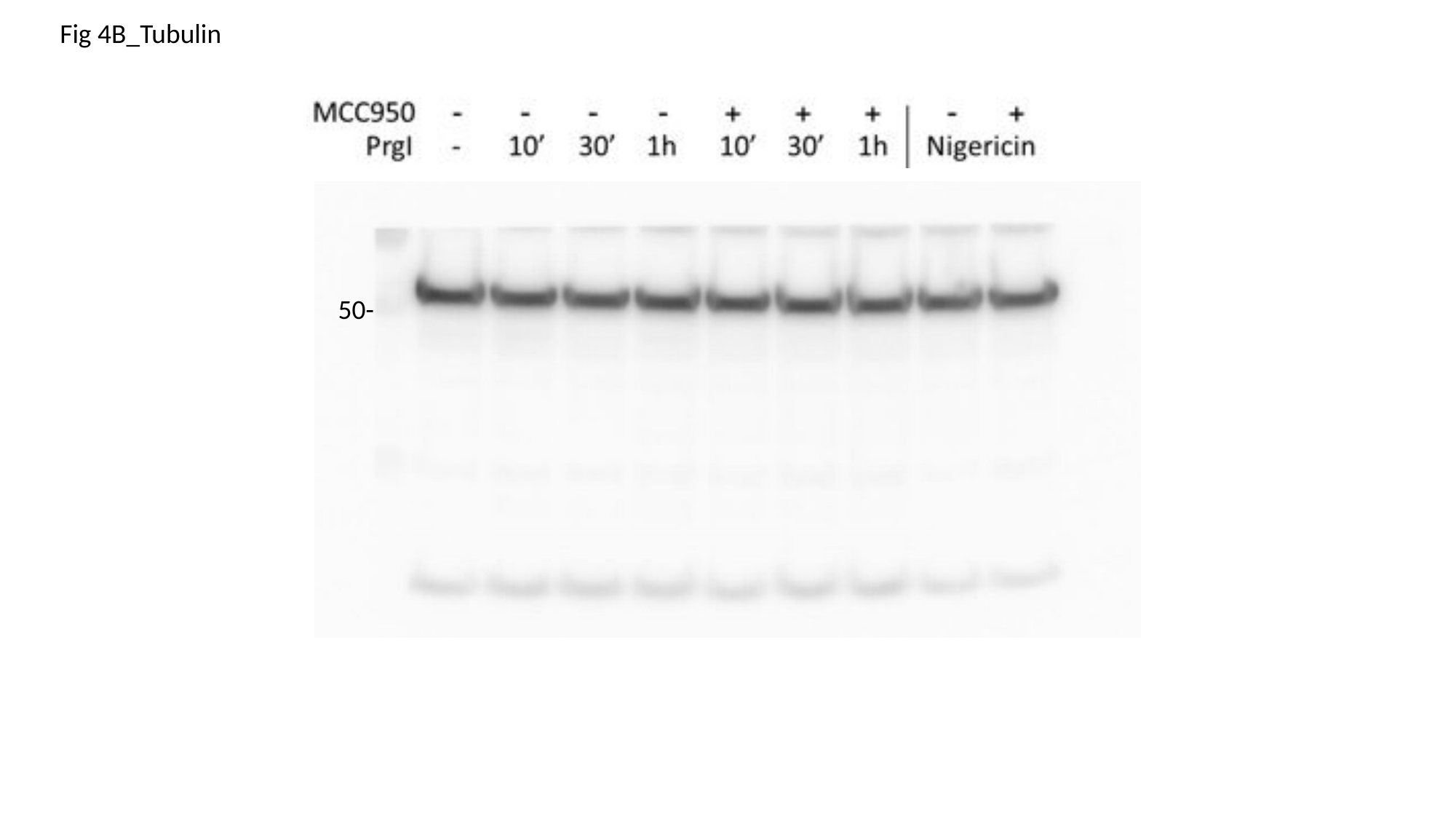

Fig 4B_Tubulin
50-

## Slide 34
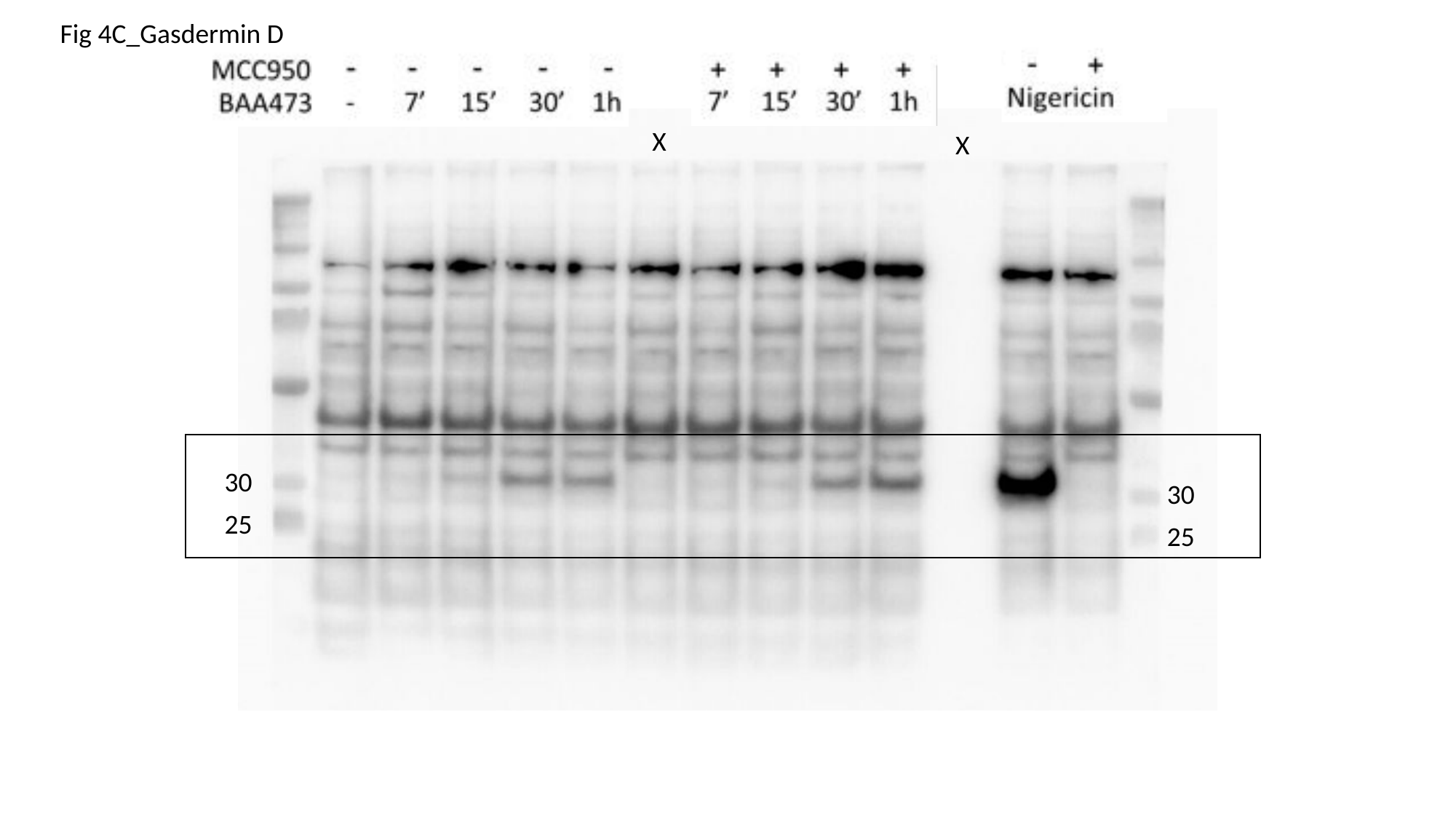

Fig 4C_Gasdermin D
X
X
30
30
25
25

## Slide 35
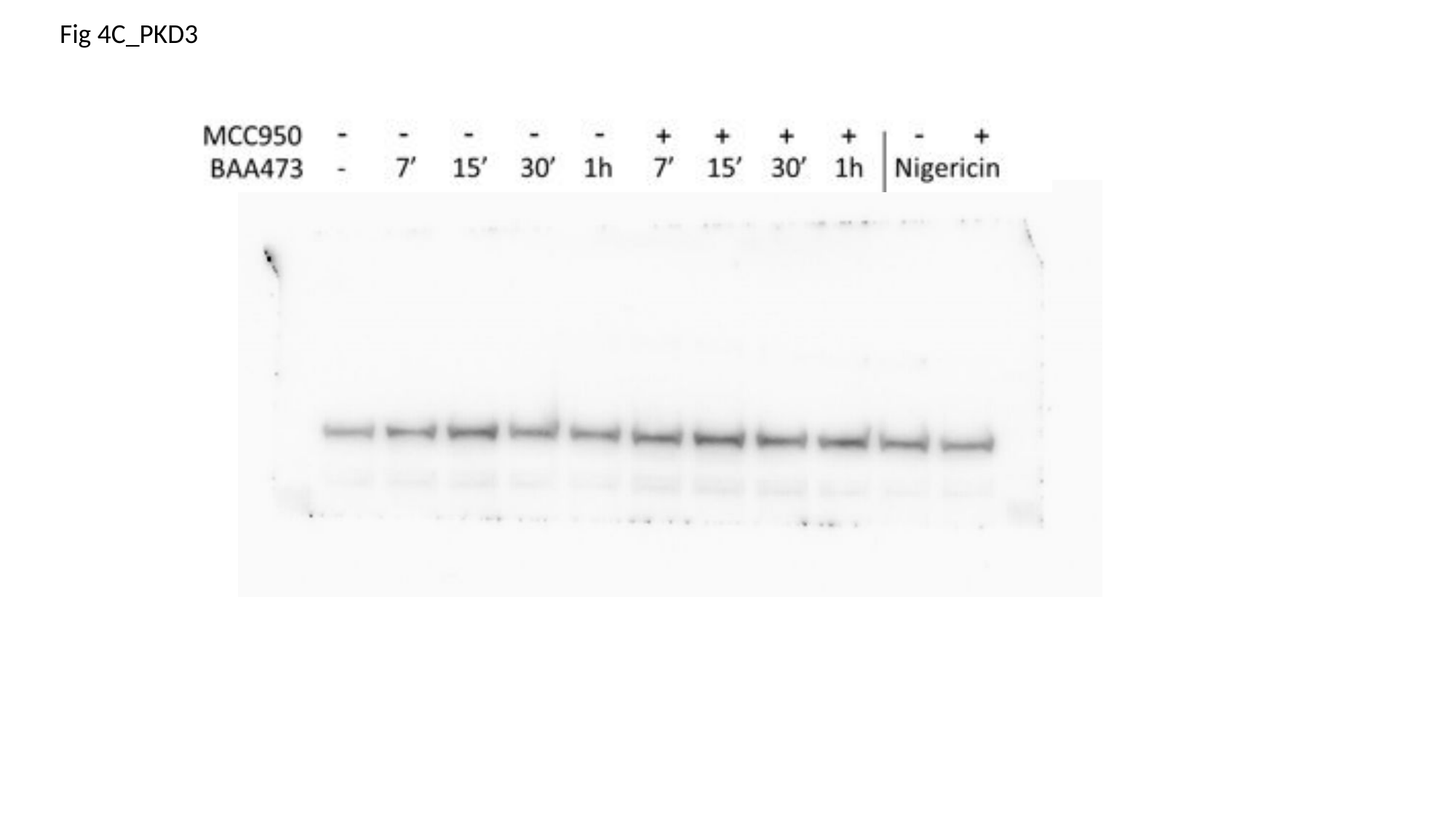

Fig 4C_PKD3

## Slide 36
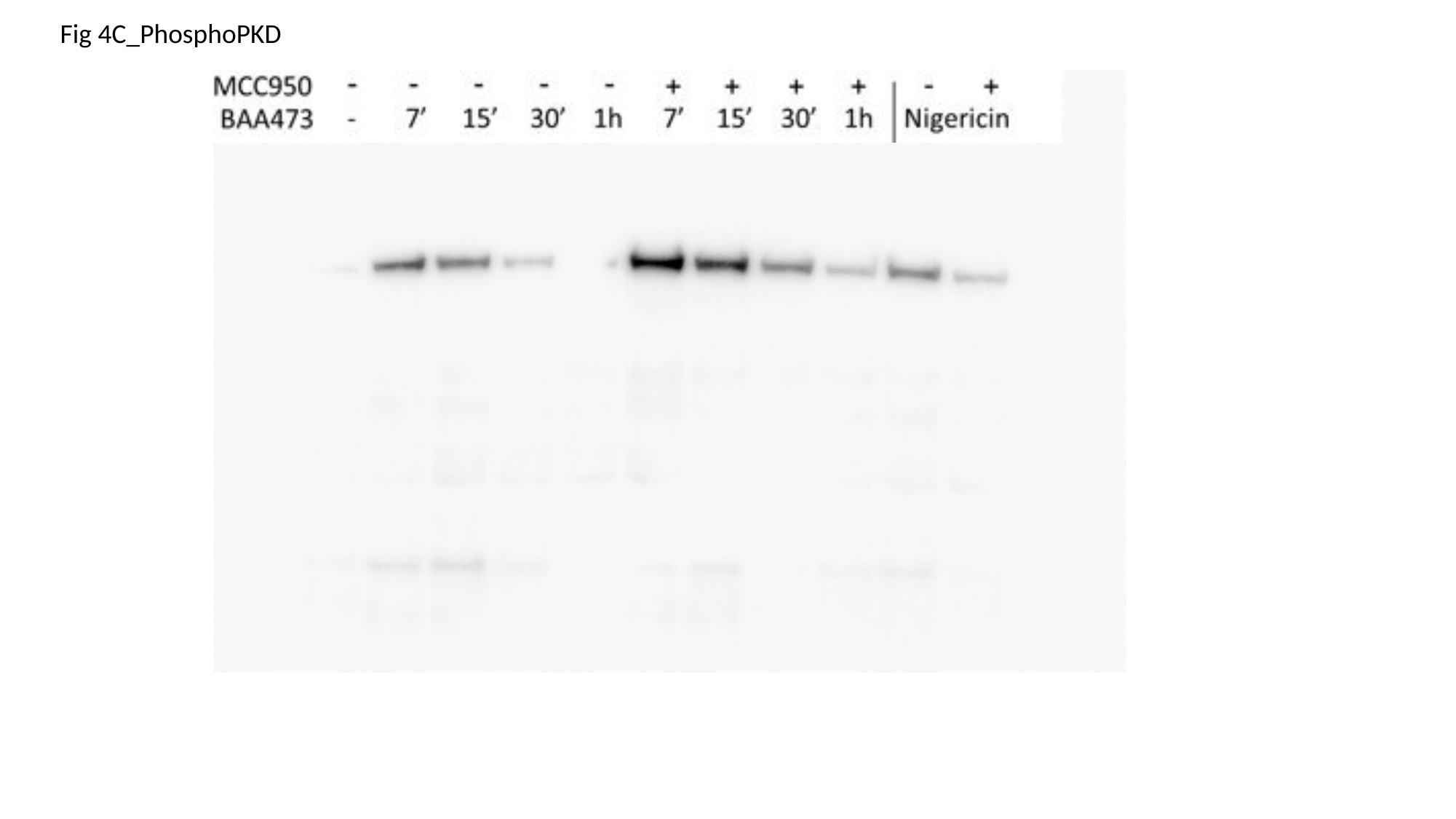

Fig 4C_PhosphoPKD

## Slide 37
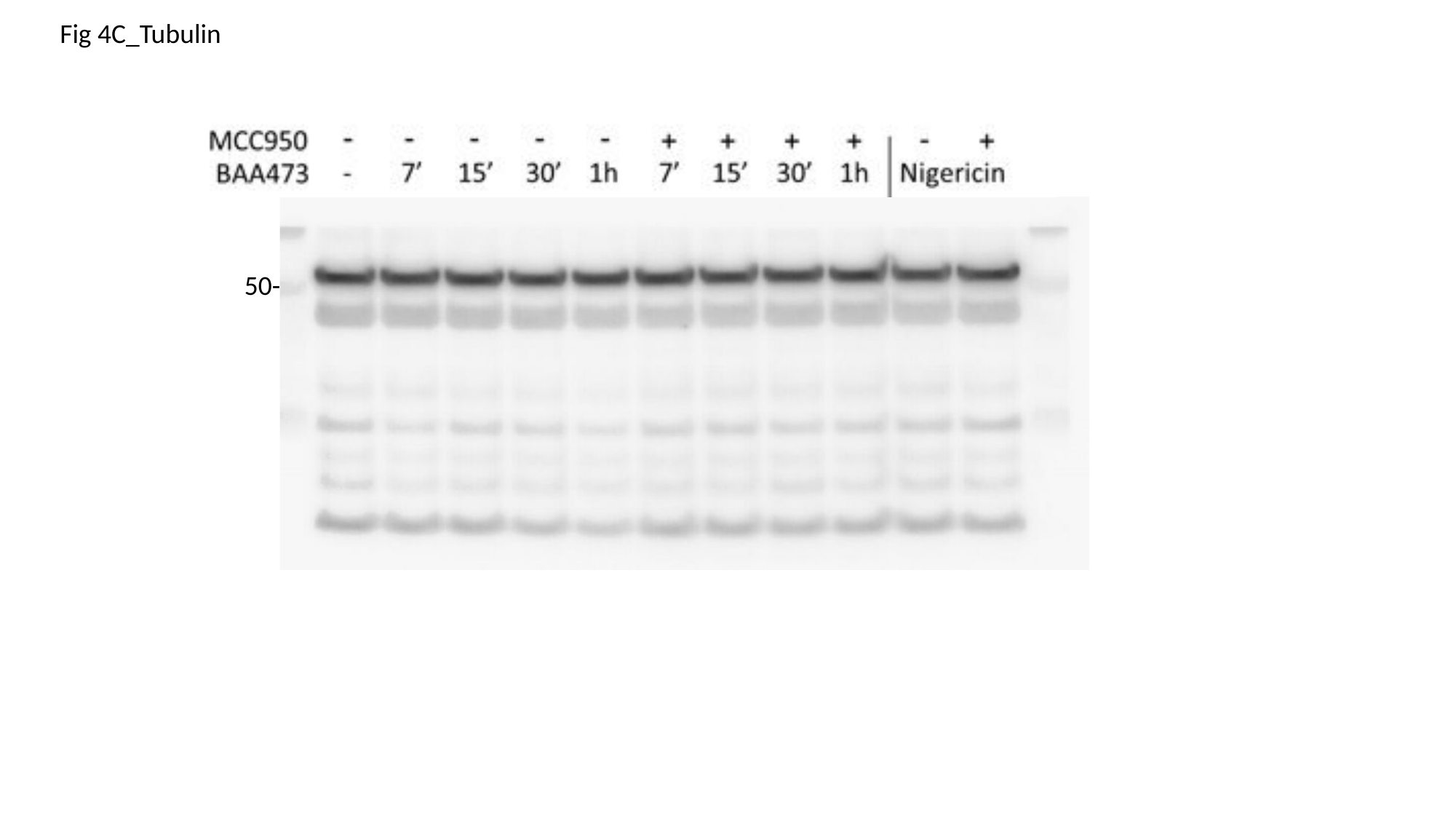

Fig 4C_Tubulin
50-

## Slide 38
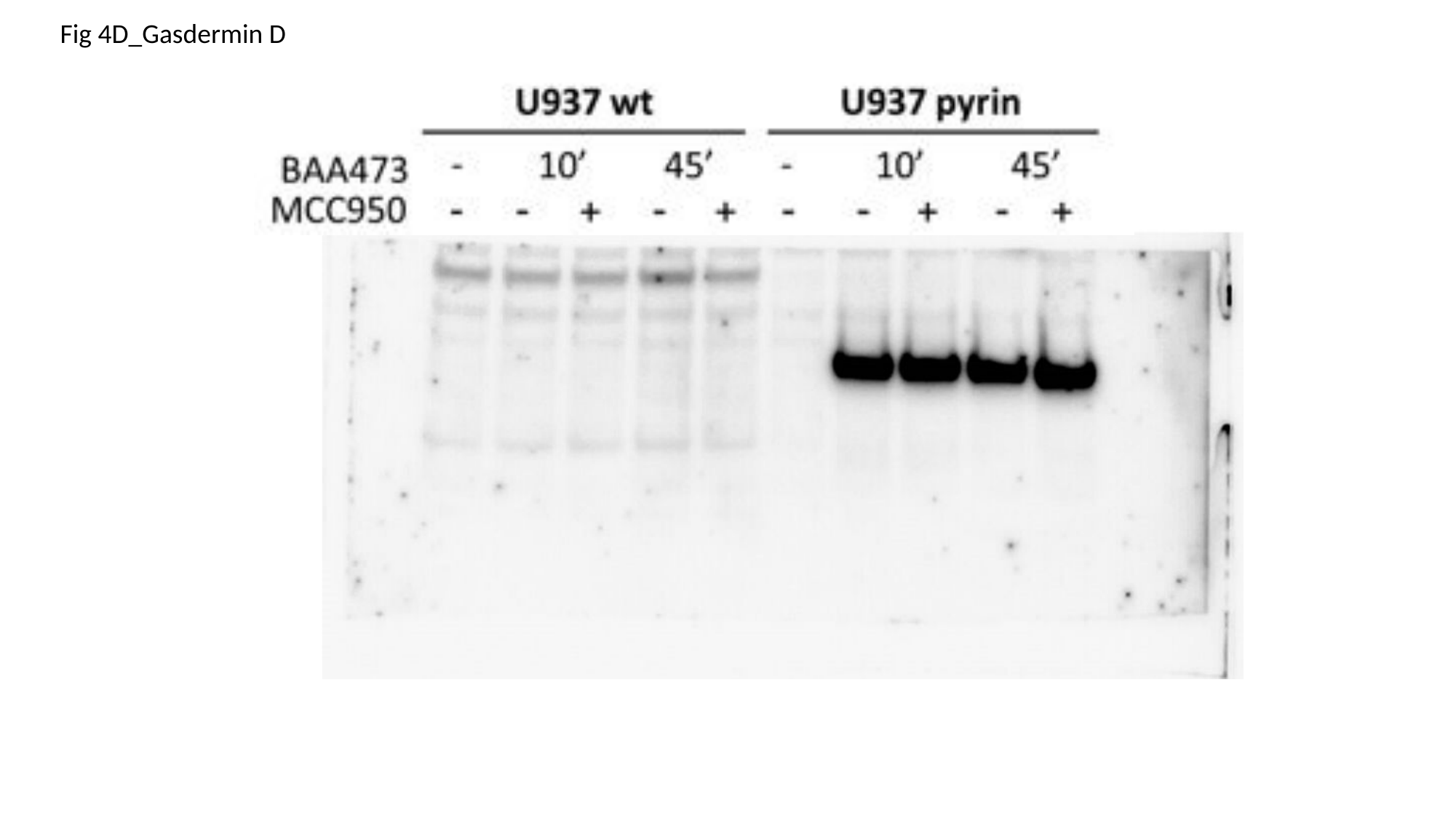

Fig 4D_Gasdermin D

## Slide 39
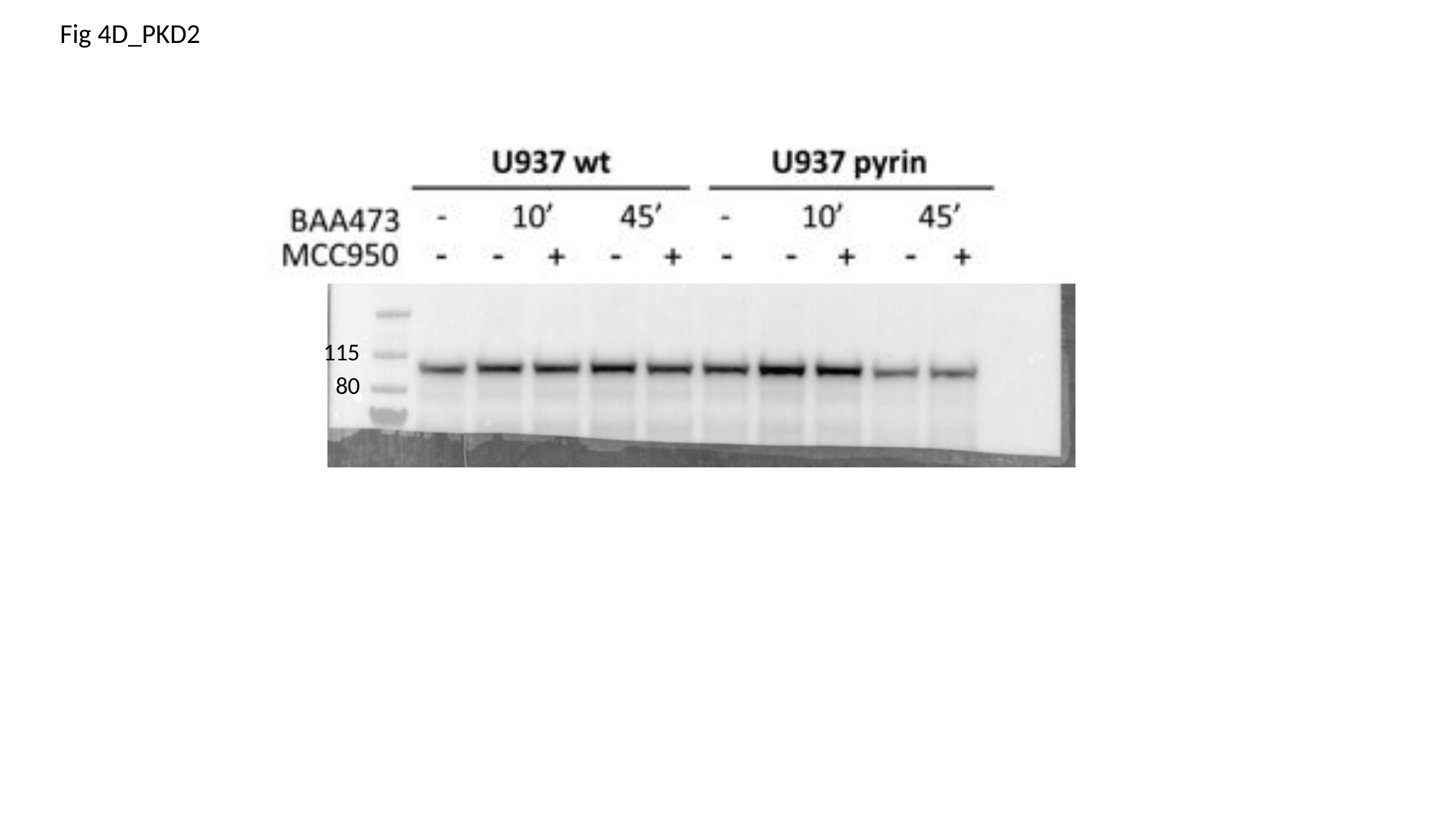

Fig 4D_PKD2
115
80

## Slide 40
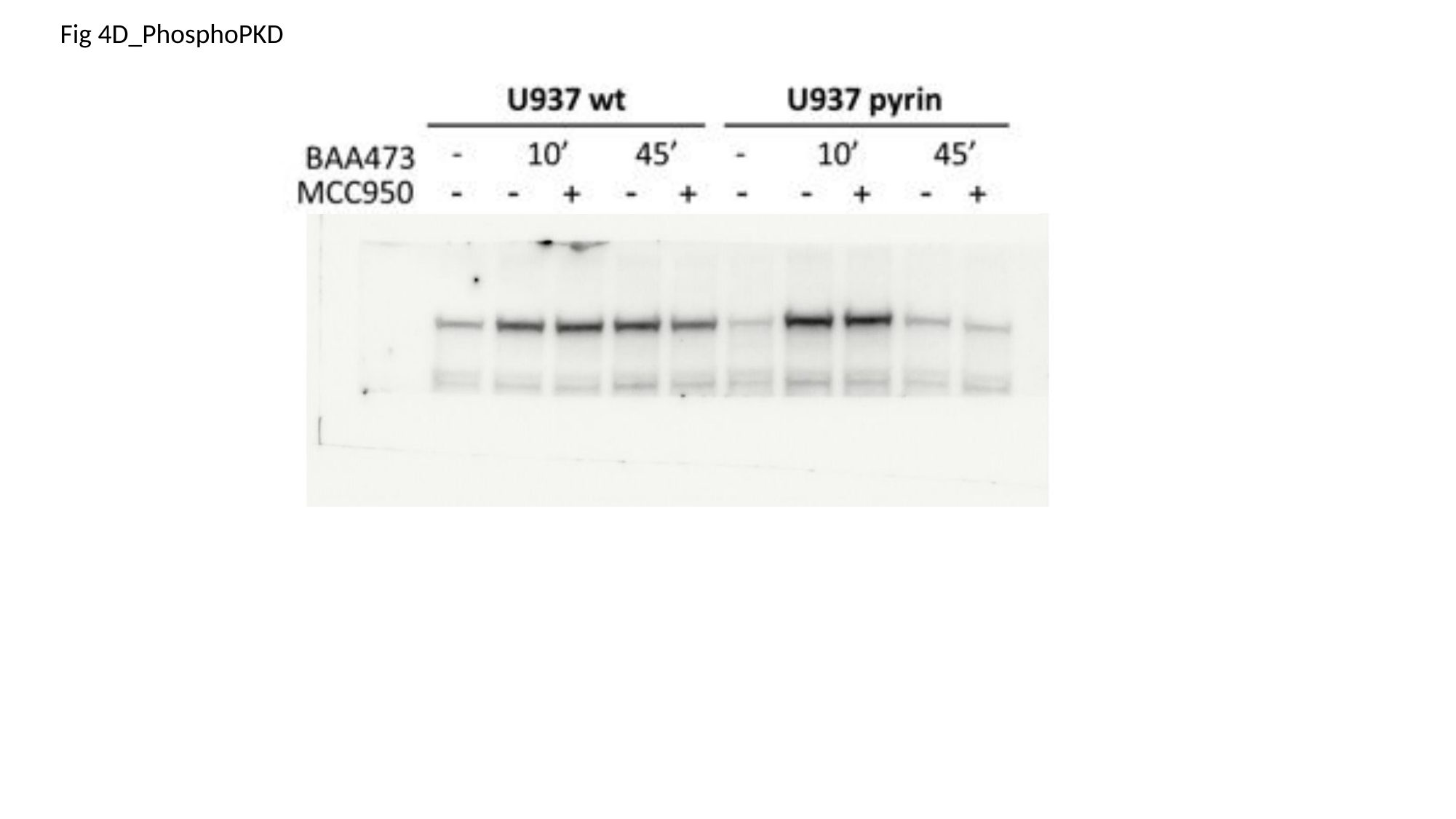

Fig 4D_PhosphoPKD

## Slide 41
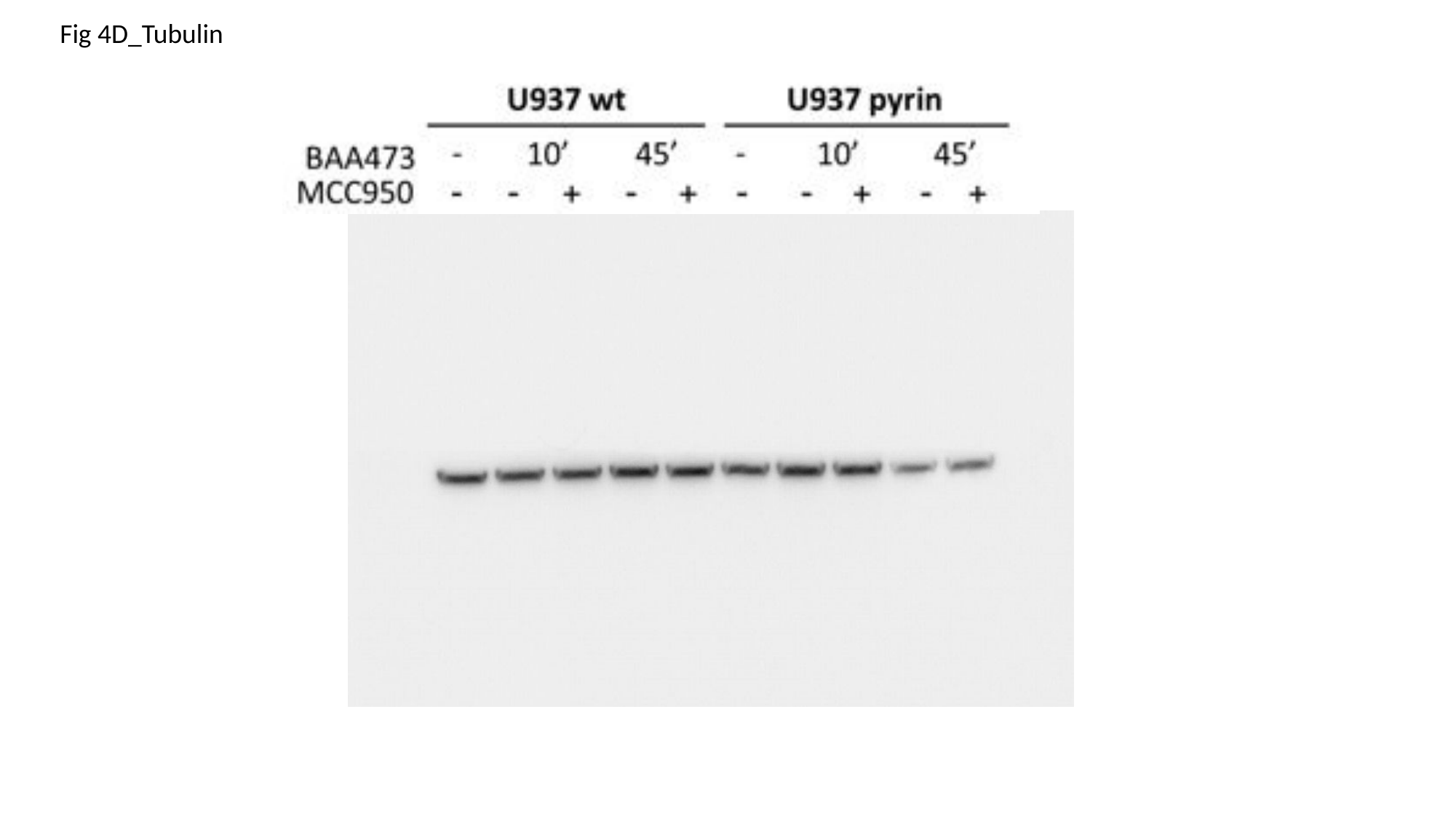

Fig 4D_Tubulin
